# Unravelling the Impact of Metal Dopants and Oxygen Vacancies on Syngas Conversion over Oxides: A Machine Learning-Accelerated Study of CO Activation on Cr-Doped ZnO Surfaces

**DOI:** 10.1021/acscatal.3c03648

**Published:** 2023-11-08

**Authors:** Yulan Han, Jiayan Xu, Wenbo Xie, Zhuozheng Wang, P. Hu

**Affiliations:** † School of Chemistry and Chemical Engineering, Queen’s University Belfast, Belfast BT9 5AG, U.K.; ‡ School of Physical Science and Technology, ShanghaiTech University, Shanghai 201210, China; § PetroChina Petrochemical Research Institute, Beijing 102206, China

**Keywords:** oxygen vacancy, Cr doping, CO activation, machine learning potential, genetic algorithm, DFT, syngas conversion

## Abstract

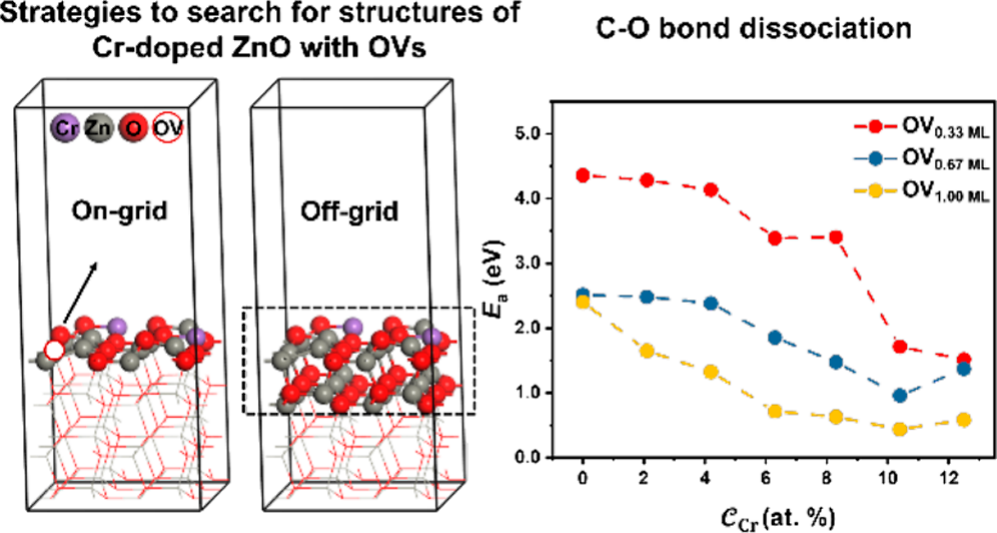

As a critical component of the OX–ZEO composite catalysts toward syngas conversion, the Cr-doped ZnO ternary system can be considered as a model system for understanding oxide catalysts. However, due to the complexity of its structures, traditional approaches, both experimental and theoretical, encounter significant challenges. Herein, we employ machine learning-accelerated methods, including grand canonical Monte Carlo and genetic algorithm, to explore the ZnO(1010) surface with various Cr and oxygen vacancy (OV) concentrations. Stable surfaces with varied Cr and OV concentrations were then systematically investigated to examine their influence on the CO activation via density functional theory calculations. We observe that Cr tends to preferentially appear on the surface of ZnO(1010) rather than in its interior regions and Cr-doped structures incline to form rectangular islands along the [0001] direction at high Cr and OV concentrations. Additionally, detailed calculations of CO reactivity unveil an inverse relationship between the reaction barrier (*E*_a_) for C–O bond dissociation and the Cr and OV concentrations, and a linear relationship is observed between OV formation energy and *E*_a_ for CO activation. Further analyses indicate that the C–O bond dissociation is much more favored when the adjacent OVs are geometrically aligned in the [1210] direction, and Cr is doped around the reactive sites. These findings provide a deeper insight into CO activation over the Cr-doped ZnO surface and offer valuable guidance for the rational design of effective catalysts for syngas conversion.

## 1 Introduction

Zinc oxide (ZnO) materials have garnered tremendous interest due to their extensive applications in the semiconductor, optical, and chemical industries. Particularly, ZnO-based catalysts (such as Zn_*x*_Cr_*y*_O_*z*_, Zn_*x*_Zr_*y*_O_*z*_, and Zn_*x*_Al_*y*_O_*z*_) have been widely utilized in various industrial processes, including water–gas shift reaction,^1^ methanol synthesis,^2,3^ and syngas conversion into light olefins.^4−7^ The direct conversion of syngas, consisting of CO and H_2_, is an intriguing process to realize light-olefin transformation, of which the selectivity of hydrocarbons is typically restricted by the Anderson–Schulz–Flory (ASF) distribution model.^8^ Recent studies have demonstrated that tandem catalysts with metal oxide–zeolite composite catalysts can effectively break the ASF distribution limitation, leading to high selectivity in direct syngas conversion into mixed light olefins. For example, Jiao et al., reported an efficient ZnCrO_*x*_–zeolite composite catalyst, named the OX–ZEO catalyst, for the direct syngas conversion, which broke the ASF distribution limit and achieved high selectivity of light olefins (94%) and only 2% methane at 17% CO conversion.^5^ Cheng et al., found that ZnO–ZrO_2_/SAPO-34 catalyst displayed 70% selectivity toward light olefins with 10% CO conversion at 1 MPa/400 °C.^9^ Additionally, Mn-based oxides mixed with SAPO-34 were also observed to exhibit superior selectivity at a CO conversion of 7%.^10^

Despite the aforementioned achievements in tandem catalytic conversions of syngas, the level of the CO conversion is typically low. It is generally agreed that CO activation occurs over the metal oxide surfaces, while C–C coupling takes place at the acid sites of the zeolite. According to Liu et al., the CO conversion depends on the metal oxide components.^11^ They demonstrated that ZrO_2_ catalysts with surface oxygen vacancies (OVs) could provide effective sites to activate CO. Similarly, the activation of CO on the ZnCrO_*x*_ catalyst was shown as a vital elementary step in the OX–ZEO process.^12^ Therefore, elucidating the active structures of metal oxide surfaces at the atomic level will offer fundamental insights into the design of new catalysts for CO conversion.

As one of the first-generation industry catalysts for syngas to methanol and a crucial ingredients of the composite catalysts toward light olefins, ZnCrO was extensively studied to link the active structures for the process.^13−16^ With the increasing Cr/Zn ratio to 1.0, the CO conversion gradually rose with the characteristic peaks of ZnO becoming weak and those of ZnCr_2_O_4_ becoming more evident.^15,17^ Previous research was primarily focused on one possible active phase, the ZnCr_2_O_4_ spinel structure. Very little is known about another potential active phase, the Cr-doped ZnO. Indeed, metal doping has been demonstrated to be an effective method of modifying the electronic properties and chemical activity of oxide materials,^18−20^ which could be essential for the activation of CO. Furthermore, surface OV sites were shown to exhibit exceptional CO activation activity, with high OV coverage being a crucial prerequisite for enhanced performance.^6,7,21^ However, little is known about how the geometric arrangement of OVs affects the activity of the surface. Thus, a comprehensive investigation of the impact of the Cr-doping effect of ZnO on the CO activation ability, with varying degrees of reduction and different OV configurations, is highly desirable.

Most theoretical studies conducted on Cr-doped ZnO were performed on simplified surface models that neglect the potential impact of surface reconstruction on the kinetics of CO activation. Typically, metal doping and surface reduction under experimental conditions are understood in terms of atomic substitution or vacancy formation within the crystal lattice. It is expected that surface reconstructions in this kind of system can readily occur. Thus, it is essential to explore the vast structural space of the ternary system to establish a connection between the atomic structure of Cr-doped ZnO and its catalytic activity. However, accurate prediction of the thermodynamically favored structures of catalysts remains a challenging task due to the relatively high computational cost of DFT calculations. To address this issue, the use of machine learning (ML)-based methods has gained popularity as they can accelerate the structure searching process with high precision.^22−28^

In this work, we chose Cr-doped ZnO(1010) [ZnO(1010) is the most stable ZnO surface] surface as a model for Cr–Zn–O ternary system to identify the stable Cr-doped ZnO(1010) surfaces with various concentrations of Cr and OV (), aiming to establish the relationship between CO activation and  and . Because the structural space of Cr–Zn–O ternary system is too large to use the DFT approach, a Zn–Cr–O ternary neural network (NN) potential was trained via an active learning scheme using the genetic algorithm (GA)-NN data set to accelerate this global structure search. Our findings reveal a preference for Cr incorporation on the ZnO(1010) surface rather than the interior of the system, with a surface reconstruction observed at high , forming Cr islands along the [0001] direction. Under the reaction conditions, the ZnO surface with 10.4%  shows the highest activity toward the C–O bond dissociation. The effective energy barrier (*E*_a_) of C–O bond cleavage decreases gradually with increasing  and . Notably, the geometric configurations of OVs play a significant role in *E*_a_; the activation of C–O bonds can be significantly enhanced when OVs are aligned along [1210] with Cr doped at the reactive center. Additionally, a linear correlation is revealed between *E*_a_ and the formation energy of OV. The obtained results provide new insights into designing a more active center in metal-doped oxide systems and strengthen our mechanistic understanding of C–O bond activation.

## 2 Methods

### 2.1 DFT Calculations

In this work, all plane-wave DFT calculations were carried out using the Vienna Ab initio Simulation Package (VASP)^29,30^ under the framework of the generalized gradient approximation with the Perdew–Burke–Ernzerhof (PBE) functional.^31^ The projector-augmented-wave pseudopotentials were used with a cutoff energy of 400 eV for the plane-wave basis expansion. Owing to the high computational cost of the hybrid functional HSE06, the PBE + *U* was used to optimize the structures because of the good compromise between the accuracy and computational cost.^32−34^ The effective Hubbard *U*_eff_ = *U* – *J* was determined by benchmarking with HSE data and was found to be 3.0 eV for Cr and Zn, which adequately described the structure and OV formation energy (*E*_OV_) for various systems containing Zn or Cr (see Section 1.1 of the Supporting Information). *E*_OV_ was selected for benchmarking due to its significance in evaluating the equilibrium  under the reaction condition, which has a great impact on the C–O bond activation. The slab was separated by a vacuum of 15 Å in the *z*-direction to ensure negligible interaction between the slab and its images. The density of states (DOS) calculations were performed using HSE06 hybrid functionals.^35^ Constrained minimization was used to search transition states (TSs) that were further verified by vibrational frequencies.^36−40^

### 2.2 ML-Accelerated Global Optimization Methods

Because the high computational cost of the traditional DFT calculations restricts the size of the available database, herein, we developed ML-accelerated global optimization methods, aiming to expedite structure optimization to expand the sample space. An NN potential was developed for the Zn–Cr–O ternary system to allow efficient structure space exploration, which was automatically constructed using an efficient iterative active learning procedure. We adopted the embedded atom NN potential (EANNP) developed by Zhang et al.^41^ This method employs an embedded atom descriptor as the atomic representation and a deep NN as the regression model. The training data set consists of different compositions and configurations generated by ab initio molecular dynamics and GA-based configurational search (see Section 1.2 of Supporting Information), comprising a total of 144 systems and 107,069 structures. These structures, being iteratively added into the data set during EANNP generation, were selected from the GA global optimization trajectories with uncertainty control and CUR selection procedures.^42,43^ The pipeline involves utilizing an open-sourced package called “Generating Deep Potential with Python (GDPy)”.^44^ With the low-energy structural candidate obtained from GA-NN, DFT calculations have been utilized to verify the most stable structures at the final stage. Unless stated otherwise, all energetics data reported in this work are from DFT calculations. GAs are metaheuristic optimization algorithms inspired by Darwinian evolution. Performing crossover, mutation, and selection operations, the algorithm progresses a population of evolving candidate solutions.^45^

In this work, EANNP uses 6 Gaussian-type radial functions with automatically learned parameters and a third-order (*L* = 3) angular expansion for the atomic representation. A cutoff of 6.0 Å is considered for neighboring atoms. The atomic-energy fitting NN uses a hidden-layer architecture of (256 × 128 × 64 × 32). The uncertainty criteria for unlearned configurations are set to 0.05–0.25 eV/atom for energy. The overall performance of EANNP on the complete data set reaches a root-mean-square error (RMSE) in the energy of 0.02 eV/atom and an RMSE in forces of 0.22 eV/Å for Cr, 0.13 eV/Å for O, and 0.10 eV/Å for Zn, respectively.

### 2.3 Grand Canonical Monte Carlo Simulation

The grand canonical Monte Carlo (GCMC) simulation was conducted on the Cr-doped ZnO(1010) surface using a unit cell of (15 × 9) containing 13 Zn–O layers and a total of 7020 atoms. Since the simulation works in the grand canonical ensemble, the chemical potential μ, volume *V*, and temperature *T* of the system are fixed. During each step of a GCMC simulation, three actions are permitted on the systems to interact with an external reservoir, namely, the exchange action for O atoms, the move action, and the swap action for Cr ions. The acceptance of every action is decided by the Metropolis MC algorithm to make the state selection according to the probability (*P*)
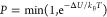
1where Δ*U* is the potential energy change after the action, *k*_B_ is the Boltzmann constant, and *T* is the simulation temperature. The potential energies of the generated configurations were evaluated by the trained NN. In this work, we considered the distribution of Cr-doped ZnO under the experimental preparation conditions of the Zn_*x*_Cr_*y*_O_*z*_ catalyst (798 K).^5^ The fixed physical quantities in the system are μ_O_, *V*, *T*, *N*_Zn_, and *N*_Cr_.

The *z* distribution function, *g*(*z*), which can give the probability of the Cr at a given distance from the bottom of the structure in the *z* direction was calculated
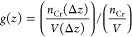
2where *n*_Cr_(Δ*z*) is the number of Cr at different heights originating from the bottom of the system and *n*_Cr_ is the number of Cr in the whole system. *V*(Δ*z*) denotes the volume in the *z* direction corresponding to the distance interval (Δ*z*), while *V* represents the total volume of the system, considering a 60 Å vacuum layer in the *z* direction.

### 2.4 Atomistic Thermodynamics

The concentration of Cr is defined as follows
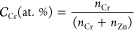
3where *n*_Cr_ and *n*_Zn_ are the numbers of Cr atoms and Zn atoms in the systems, respectively.

The concentration of surface OV is defined as follows
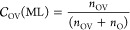
4where *n*_OV_ and *n*_O_ are the numbers of OV and O atoms on the outmost layer [the unit is monolayer (ML)], respectively.

Δ*G*_Cr_ can be used to obtain the required free energy for the substitution of Cr for Zn at various , and is calculated as follows
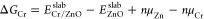
5where μ_α_ represents the atomic chemical potential of element α, μ_Zn_ is determined by μ_Zn_ = μ_ZnO_^bulk^ – μ_O_, and μ_Cr_ is determined by the formula . Here μ_ZnO_^bulk^ and  denote the energy of the ZnO/Cr_2_O_3_ unit in its bulk state. *E*_Cr/ZnO_^slab^ and *E*_ZnO_^slab^ are the total energies of the Cr-substituted Zn(1010) and the pristine Zn(1010).

Previous works have demonstrated the presence of several defects on the ZnO(1010) surface, such as OVs, Zn vacancies, Zn–O pair vacancies, and others.^18,46−48^ Supposing that the Zn–O pair vacancies are exposed on the surface with a total energy of *E*_ZnO_^slab_defect^, the corresponding Gibbs free energy of formation for Cr in the Zn lattice became
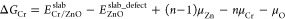
6μ_O_ is calculated in eq 7.
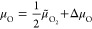
7where Δμ_O_ contains all the temperature- and pressure-dependent free energy contributions. Given that the high-spin ground state of the oxygen molecule is poorly described in DFT calculations, the method to calculate the free energy of the O_2_ (μ̃_O_2__) molecule was derived according to μ̃_O_2__(g) = 2μ̃_H_2_O_(l) – 2μ̃_H_2__(g) + 4 × 1.23 (eV) under standard conditions. According to the reaction conditions, we used a temperature of 673 K and a pressure of 2.5 MPa (H_2_/CO = 1.5). Δμ_O_ = −3.3 eV when the surface is in equilibrium with CO (see Section 1.3 of Supporting Information). Note that negative values signify the stability of the defect surface in comparison to the perfect surface.

The formation energy *E*_OV_ of the OV is calculated by the following formula
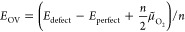
8where *E*_perfect_ and *E*_defect_ are the total energies of the supercell before and after OV generation, *n* is the number of OVs.

It is expected that the surface structure will evolve under the reaction conditions of syngas conversion in a reduction atmosphere with high temperature and pressure. The equilibrium  is evaluated by
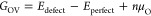
9

The adsorption energy (*E*_ads_) of adsorbates on the Zn–Cr–O surface was calculated as the energy difference between the adsorbate–Zn–Cr–O complex and the sum of the isolated Zn–Cr–O and adsorbates. The reported binding energies correspond to the most energetically favorable configurations obtained by relaxing the adsorbates from various initial structures.

The effective reaction barrier (*E*_a_) here is utilized to quantify the reaction activity, defined as follows
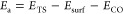
10where *E*_TS_, *E*_surf_, and *E*_CO_ are the energies of C–O bond cleavage TS, clean catalysts surface, and CO molecule, respectively.

### Strategies to Search for Structures of Cr-Doped ZnO(1010) Surfaces with Various  and 

2.5

**Figure 1:**
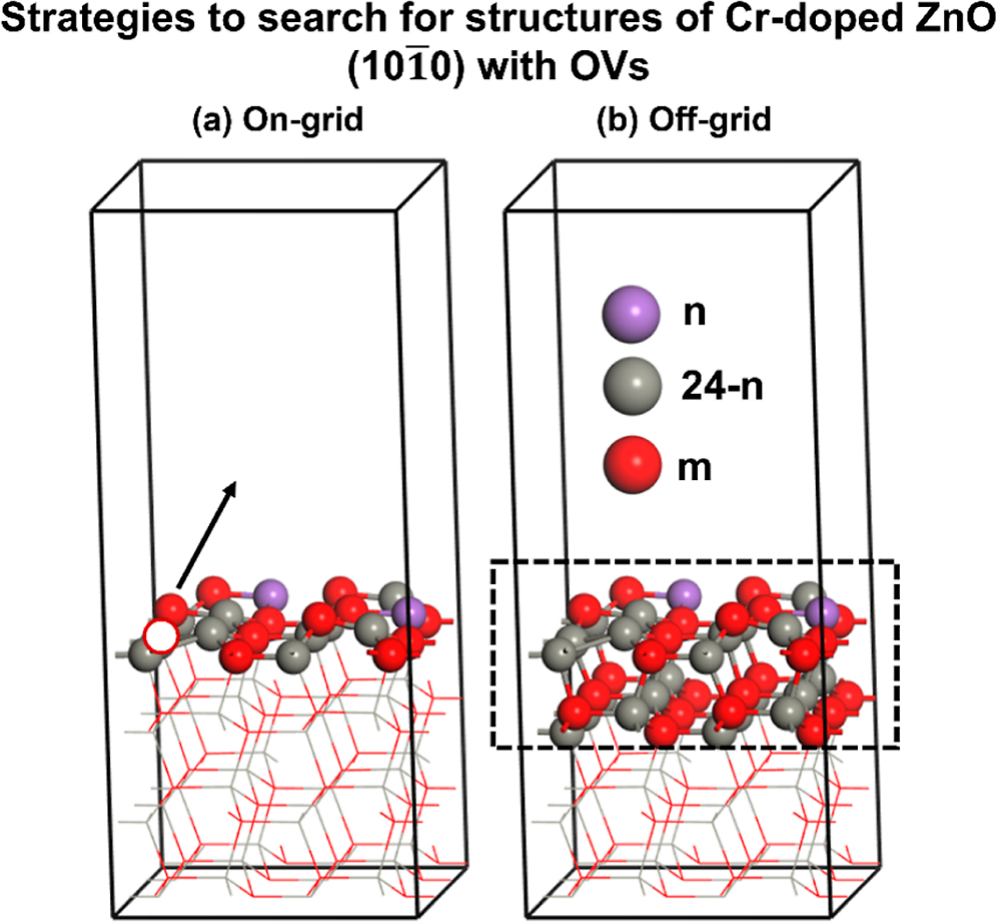
(a) On-grid strategy: the sequential removal of the O atoms one by one on the most stable Cr-doped ZnO (1010) surface identified. (b) Off-grid strategy: the GA explored the configuration of Zn_24–*n*_Cr_*n*_O_*m*_ with the top two atomic layers shown in the dashed rectangle. The third layer is relaxed, while the fourth layer is fixed during the optimization process. Color code: gray, red, and purple represent Zn, O, and Cr, respectively.

ML-accelerated GA simulations were conducted on a small ZnO(1010) surface unit cell (3 × 2) containing 4 Zn–O layers to identify the most stable surface as a function of  (ranging from 0 to 12.5%: 2.1, 4.2, 6.3, 8.3, 10.4, and 12.5%) and  (ranging from 0.00 to 1.00 ML: 0.17, 0.33, 0.50, 0.67, 0.83, and 1.00). The upper limit 12.5% for  represents a scenario where all the Zn atoms on the outermost layer are replaced by Cr atoms, while 1.00 ML  corresponds to the removal of all the O atoms on the outermost layer. To determine the Cr-doped ZnO(1010) surfaces of various , the first step was to calculate the surface structures of various  without any OVs based on the GA-EANN method. Subsequently, the Cr-doped ZnO surfaces underwent reduction by reacting with CO to form CO_2_ under the syngas conditions. Two structure searching methods to explore the surfaces with OVs were considered in this work. The first, referred to as the on-grid strategy, involves the sequential removal of O atoms on the surface of the most stable Cr-doped ZnO surface identified in the first step (see Figure 1a). This sequential removal process corresponds to the local surface optimization. The second, referred to as the off-grid strategy, involves the rediscovery of the most stable configurations of various Zn/Cr/O ratios at different  based on the GA-EANN method (see Figure 1b). This approach enables global surface optimization by exploring a broader structural space. The structures examined by these two methods are defined as unreconstructed and reconstructed structures in this work, respectively, and both are likely to appear under the reaction conditions. Hence, we investigated them separately.

## 3 Results and Discussion

Our work was structured as follows: First, we used the ML-accelerated GCMC and GA simulations for ZnO(1010) to explore the Cr-doped ZnO surfaces with various  and . Then we quantitatively investigated the C–O bond activation on these surfaces, gaining an in-depth understanding of how the concentration and arrangement of OV and Cr influence the reaction.

### 3.1 Thermodynamics of the Cr-Doped ZnO(1010) Surfaces

#### 3.1.1 ML-Accelerated GCMC Simulations on the Large Unit Cell (15 × 9) Cr-Doped ZnO(1010) Surfaces

**Figure 2:**
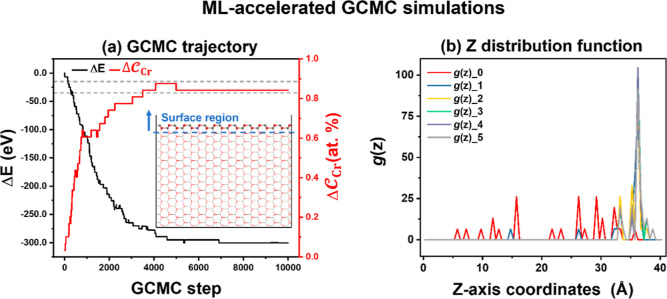
(a) Changes of the total energies (black line) and  (red line) of the surface region (*z*-coordinate greater than 34 Å, as shown in the inset figure) as a function of NN accelerated GCMC simulation step for ZnO(1010) with 1%  at 798 K. (b) Cr distribution function in the *z* direction [*g*(*z*)] for the structures obtained from GCMC simulations. *g*(*z*)_0 refers to *g*(*z*) in the original structure, and subsequent *g*(*z*) analyses were conducted at intervals of 2000 simulation steps.

Five initial ZnO(1010) surfaces with 1%  on a large ZnO(1010) surface unit cell (15 × 9) were arbitrarily generated, and then independent GCMC simulations were carried out using the MLPs under the experimental preparation conditions of the Zn_*x*_Cr_*y*_O_*z*_ catalyst (798 K)^5^ to make the results statistically reliable. Herein, one surface was selected for detailed analyses, while the remaining surfaces were presented in Figures S3–S5. In total, 10,000 steps were performed to ensure the convergence of the GCMC simulation. Figure 2a shows that the energies of the converged structures decrease with the increasing  on the surface: After the 4500th step, almost all of the Cr ions move to the vicinity of the top layer; no more changes in both the energies and the Cr ratio on the surface occur in our GCMC simulations, indicating convergence. To gain deeper insights into the distribution of Cr ions, the distribution function of Cr along the *z*-axis between 0 and 38 Å, which gives the probability of finding the Cr ions at a given distance, was computed (see Figure 2b). During the GCMC simulation, Cr ions were observed at various height in the initial structure (i.e., a relative uniform distribution). However, as the simulation progressed, the likelihood of finding Cr ions in the bulk of the structure decreases gradually, with almost all of the Cr ions eventually being located on the surface with a coordination number of 4–6 under each given condition. These results reveal a preference for Cr to localize on the surface of ZnO(1010) rather than stay in the bulk. Subsequently, we employed a smaller cell (3 × 2) to conduct a more detailed investigation into the structure and activities of the Cr-doped ZnO(1010) surface, which was identified using the GA global optimization method.

#### 3.1.2 ML-Accelerated GA Simulations on the Small Unit Cell (3 × 2) of Cr-Doped ZnO(1010) Surfaces

**Figure 3:**
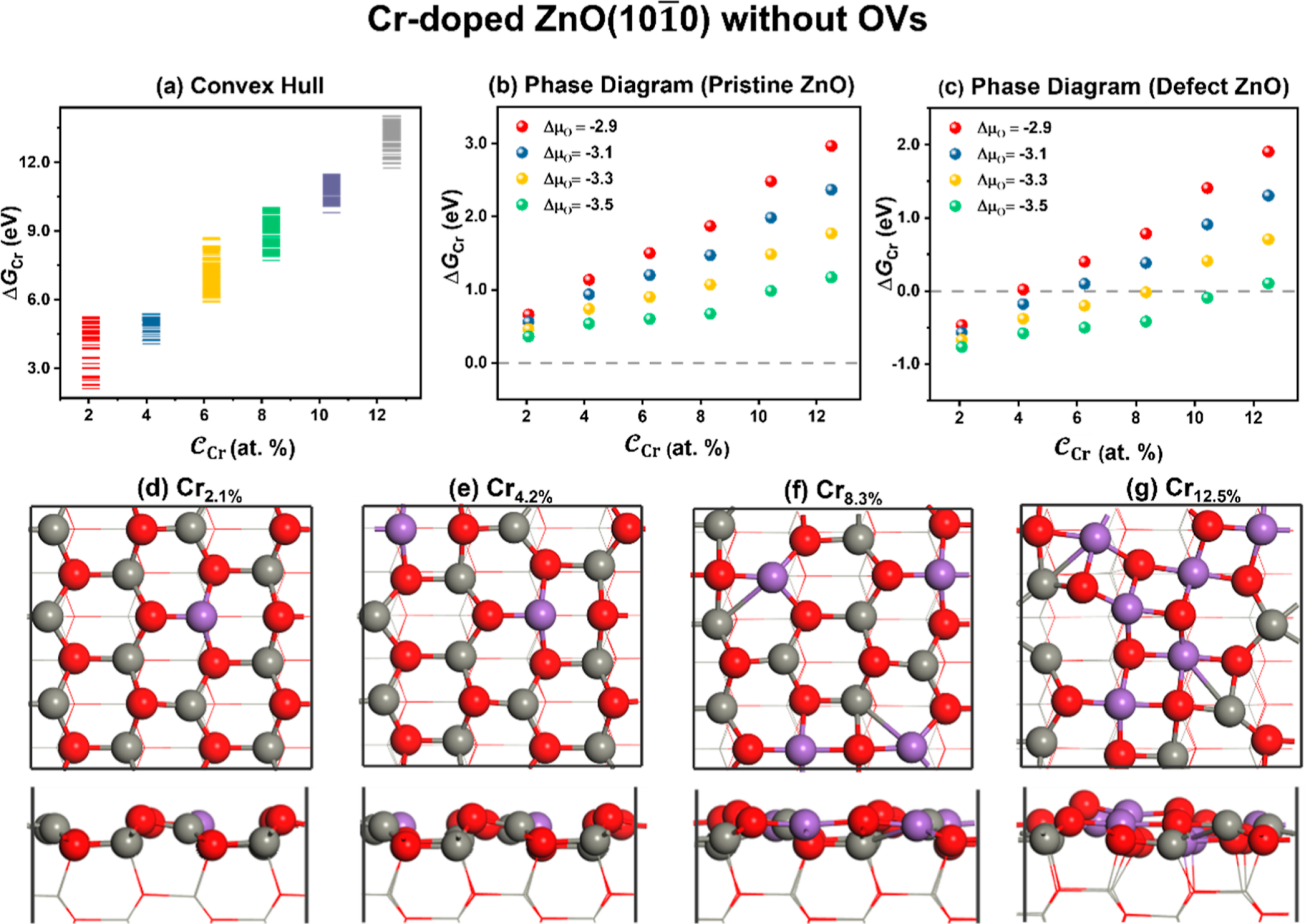
(a) Thermodynamic convex hull diagram for the Cr-doped ZnO(1010) surface referring to a gaseous O_2_ molecule and the pristine ZnO surface. (b,c) Phase diagrams made up of the most stable structures at different μ_O_ with respect to the pristine ZnO surface and the defective ZnO surface with a Zn–O pair vacancy, respectively. (d–g) Structures of the global minimum of Cr-doped ZnO with different .

The GA global optimization, based on EANN, was applied to determine the thermodynamics of Cr-doped ZnO(1010) surface phases without any OVs. A total of over 10^6^ structures were explored for all Cr-doped ZnO surfaces. The 150 most stable structures under each  were then further optimized by DFT, forming the thermodynamic convex hull for the Cr-doped ZnO(1010) surfaces with reference to μ_O_ under the standard conditions (298.15 K, 0.1 MPa) (see Figure 3a). The figure illustrates that free energy of Cr doping (Δ*G*_Cr_) increases with increasing , indicating that the preparation of Cr-doped ZnO with higher Cr doping levels is progressively difficult.

We replot the thermodynamic convex diagram to understand the stability trend of Cr-doped ZnO under a variety of Δμ_O_ values (−2.9 to −3.5 eV) in Figure 3b. The Δ*G*_Cr_ values of the Cr-doped ZnO(1010) surfaces with respect to the perfect surface under the O-rich condition are lower than those under the O-poor condition. The positive value of Δ*G*_Cr_ indicates that the experimental preparation of Cr-doped ZnO surfaces from defect-free, pristine surfaces is thermodynamically challenging. In addition, the formation energy of the Cr-doped ZnO(1010) surface referring to the defective ZnO surface containing a Zn–O vacancy is shown in Figure 3c. Compared to the trend with respect to the intact surface, the trend from the Cr-doped defective ZnO surface remains almost unchanged with the increased . Notably, Cr doping is feasible across a wide range of μ_O_, as supported by experimental studies that Cr-doped ZnO(1010) surfaces can be fabricated experimentally.^18,49^Figures 3d–g and S6 show the global minimum structures of the ZnO surface with different : Cr tends to replace the surface three-coordinated Zn atoms and pulls the nearby O atoms closer to form a 4-fold-coordinated structure. As the  increases, the arrangement of Cr tends to bond with as many O atoms as possible. When  reaches 12.5%, the surface undergoes a significant deformation, similar to the cubic structure observed in the ZnCr_2_O_4_.^15,17^ Some Cr ions exhibit a coordination number of five, which is higher than the ordered structure observed at  below 12.5%.

**Figure 4:**
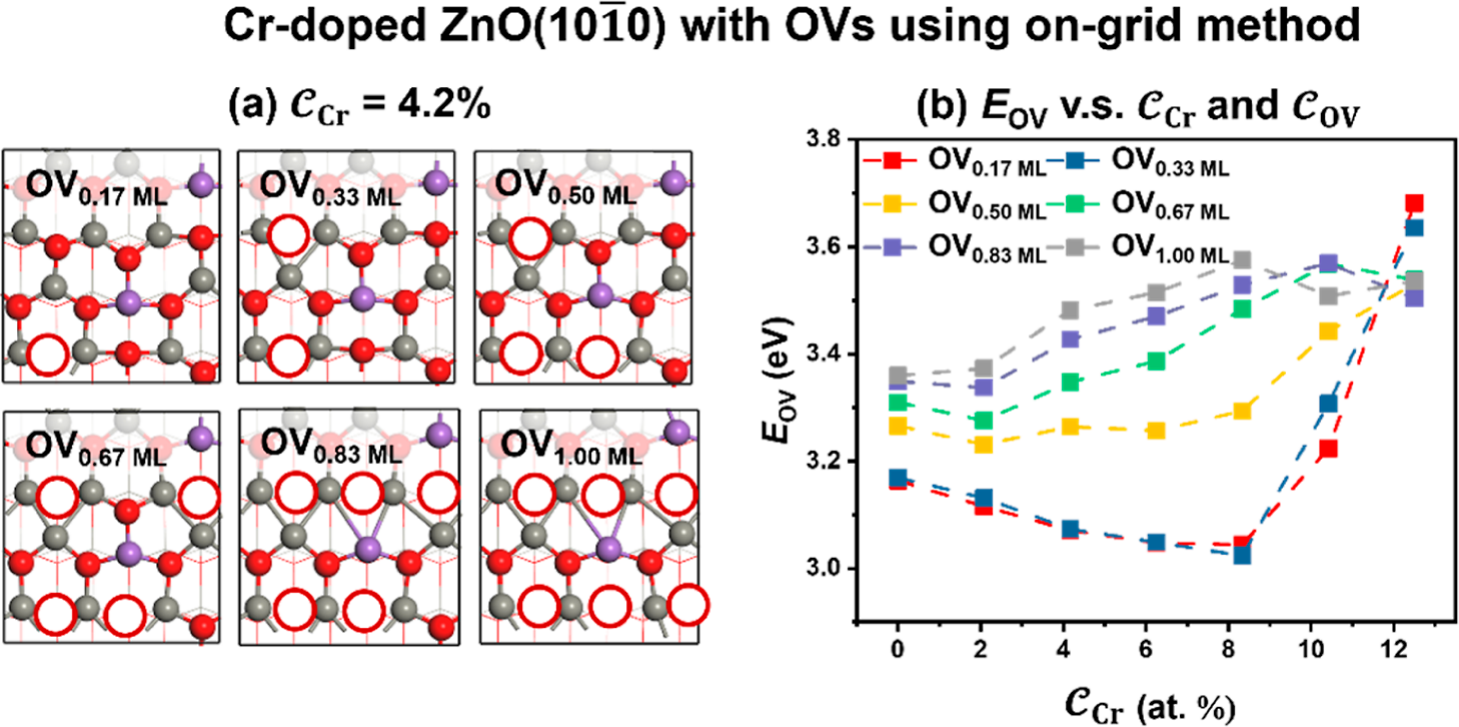
(a) Evolution of ZnO(1010) with 4.2% Cr structures at different . (b) *E*_OV_ with various  and  based on the on-grid strategy.

Then we investigated the structures of the surfaces with different reduction degrees and their stabilities. Taking ZnO(1010) with a 4.2%  as an example, the most stable structure with different  identified by the on-grid method is shown in Figure 4a. As the reduction degree is increased, the presence of the surface vacancies distorts the neighboring lattice region to form metal–metal bonds. The topmost layer of three-coordinated atoms near Zn ions is found to be the first to be reduced, followed by O near Cr, and the O directly connected to Cr show the least tendency to be reduced. We also calculated the effect of  and  on the OV formation energy (*E*_OV_) (see Figure 4b). With the increase of , the ability of the structure with  (0.17–0.83 ML) to generate OVs first increases and then decreases, indicating that the Cr doping favors the departure of the O near Zn but not the O near Cr. The Cr doping gives rise to deformation of the surface structures, and Cr ions can stabilize the extra electrons generated by the departure of the oxygen, as can be seen from the curve of projected DOSs (PDOS) in Figure S7, both of which promote OV generation around Zn ions. Meanwhile, the chemical bond between Cr and O is excessively strong, impeding the further formation of OVs.

**Figure 5:**
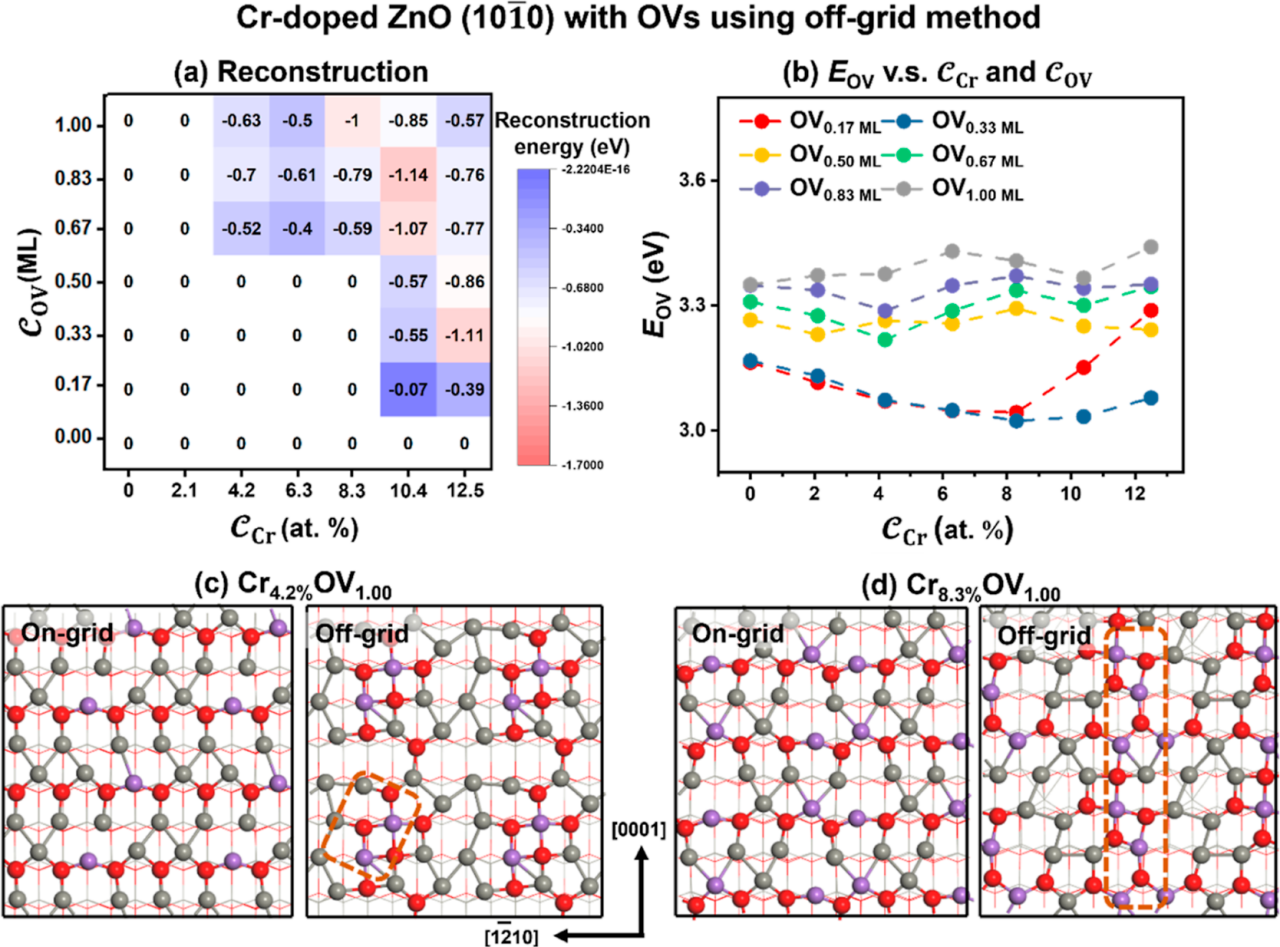
(a) Energy differences between the most stable structures produced by the off-grid and on-grid methods. The number 0 implies that the most stable structures identified by both methods are the same. (b) *E*_OV_ of the structures with various  and  based on the off-grid strategy. The most stable structures of ZnO surfaces with Cr_4.2%_OV_1.00_ (c) and Cr_8.3%_OV_1.00_ (d) were identified by on-grid and off-grid strategies.

Likewise, the most stable configurations with different  and  were identified using the off-grid strategy mentioned above. As shown in Figure 5a, the surfaces can significantly reconstruct, as indicated by the decline of the reconstruction energies (−0.07 to −1.14 eV) as the  and  increase. Conversely, the surfaces with low  and  hardly undergo reconstruction. The *E*_OV_ values of the reconstructed structures were also calculated (see Figure 5b). Compared to the structures identified by the on-grid strategy, a similar trend can be seen with the increased . However, the *E*_OV_ of the reconstructed surface under the same composition is lower from the off-grid method. Figures 5c,d and S8 show the structural comparison of the surfaces before and after reconstruction with 1.00 ML OVs. On the unreconstructed surface, the coordination number of Cr is observed to be two. However, upon reconstruction, the coordination number of Cr increases, ranging from 2 to 5. Notably, at lower , a localized stripe region with 4-fold-coordinated Cr is visible. As  increases, the tendency of Cr ions to aggregate and form stripe structures along the [0001] direction becomes prominent, which is consistent with the experimental report.^18^ This aggregation involves the simultaneous replacement of adjacent three-coordinated and 4-fold-coordinated Zn atoms by Cr, resulting in enhanced bonding with oxygen atoms and improved structural stability.

### 3.2 CO Activation

The CO activation process, which involves CO adsorption and C–O bond dissociation, plays a crucial role in syngas conversion; it was found to be the rate-determining step on ZnCr_2_O_4_ surfaces for syngas conversion.^12^ In this section, we examined the effects of Cr and OV on this key step. The CO activation primarily involves two mechanisms: the direct cleavage of the C–O bond and the H-assisted C–O bond cleavage. The barrier for each H-assisted C–O bond cleavage is significantly lower than that for the direct C–O bonding breaking while still following the same trend.^48^ In this study, we focused on the relatively simpler direct dissociation of C–O bonds to study the activity across a range of catalysts. First, the equilibrium surfaces of ZnO with varying  under the reaction condition (Δμ_O_ = −3.3 eV) were investigated to determine the optimal Cr doping ratio for efficient CO activation. Subsequently, all the structures were studied to disclose the quantitative relationship of CO activation on the ZnO surfaces with various  and . Furthermore, we analyzed the impact of the distribution of Cr and OV on the C–O bond dissociation process to gain a deeper understanding of the contributions of Cr and OV to CO activation.

#### 3.2.1 CO Activation on Cr-Doped ZnO(1010) under the Reaction Condition (Δμ_O_ = −3.3 eV)

**Figure 6:**
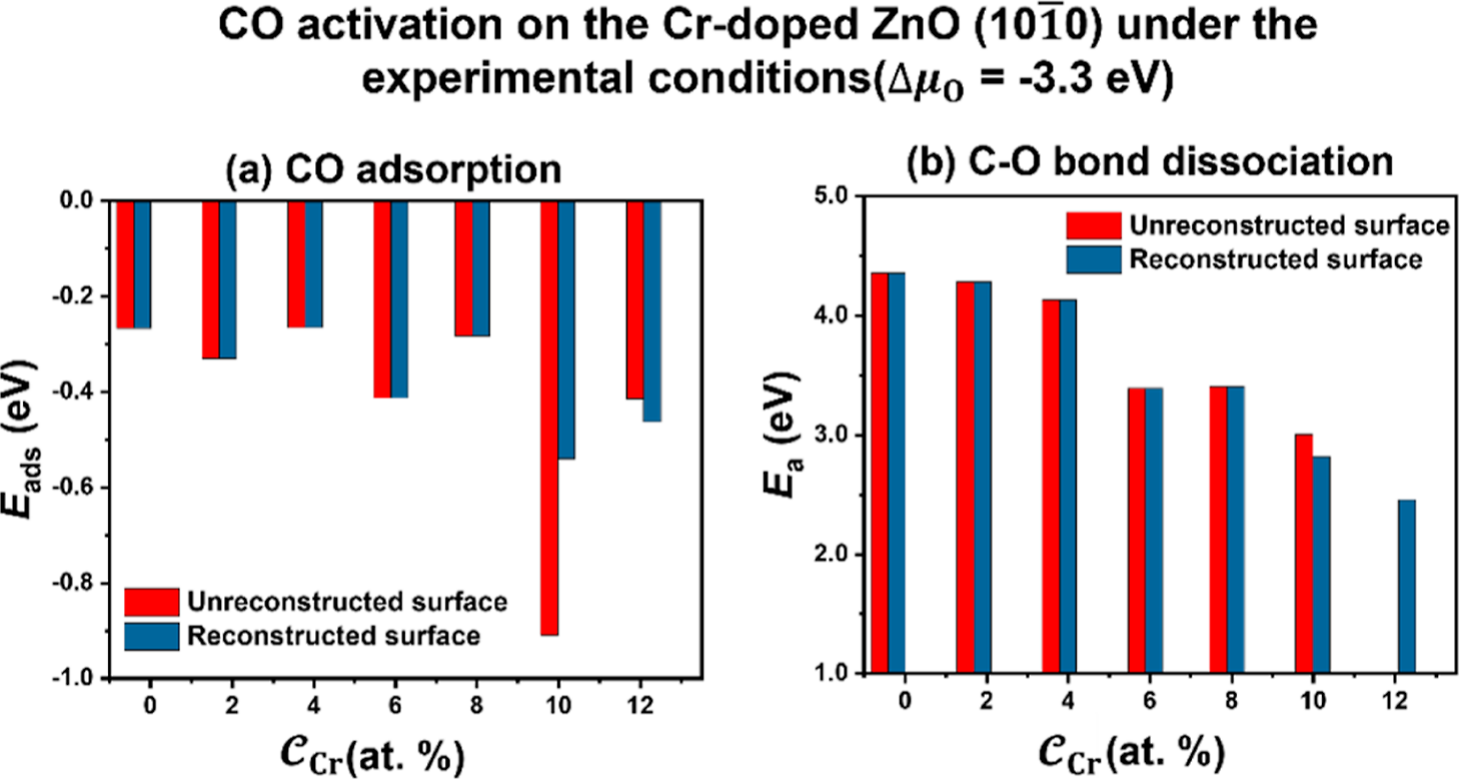
Changes of CO adsorption energies (a) and C–O bond dissociation *E*_a_ (b) on the structures with the increasing  of the equilibrium  under the syngas conversion conditions.

After taking into account the reaction conditions (Δμ_O_ = −3.3 eV), the equilibrium  identified by on-grid method is 0.33 ML for the surfaces with 0.0 to 8.3% , 0.17 ML for the surface with 10.4%  and 0.00 ML for the surface with 12.5% , as can be seen in Figures S9, S10 and S11a. By contrast, the equilibrium  determined by the off-grid method is 0.33 ML for all  in Figure S11b. Clearly, the structures with  (0.0–8.3%) calculated by both methods are the same. With the knowledge of the surface structures under the reaction conditions, we can now discuss the results of adsorption and dissociation of CO on these equilibrium surface structures. The unreconstructed surface exhibits the highest adsorption energy of CO at a  of 10.4%, while the reconstructed surface displays relatively high adsorption energies at  greater than 8.3% (see Figure 6a). Furthermore, Figure 6b illustrates the activation energies (*E*_a_) for the dissociation of the C–O bond on the ZnO surfaces with different  under the reaction conditions. Generally, as  increases, *E*_a_ gradually decreases, except for the structure with 12.5%  identified by the on-grid method. Notably, the active sites were absent on the unreconstructed surface with 12.5%  due to the inability to produce OV, thereby impeding the breakage of the C–O bond. Both the on-grid and off-grid structure search methods identified a surface exhibiting relatively high activity at a  of 10.4%. Thus, the ZnO surface with 10.4%  appears to exhibit the highest activity under the reaction conditions.

#### 3.2.2 Impact of Cr and OV Concentrations on CO Activation on Cr-Doped ZnO(1010) Surfaces

**Figure 7:**
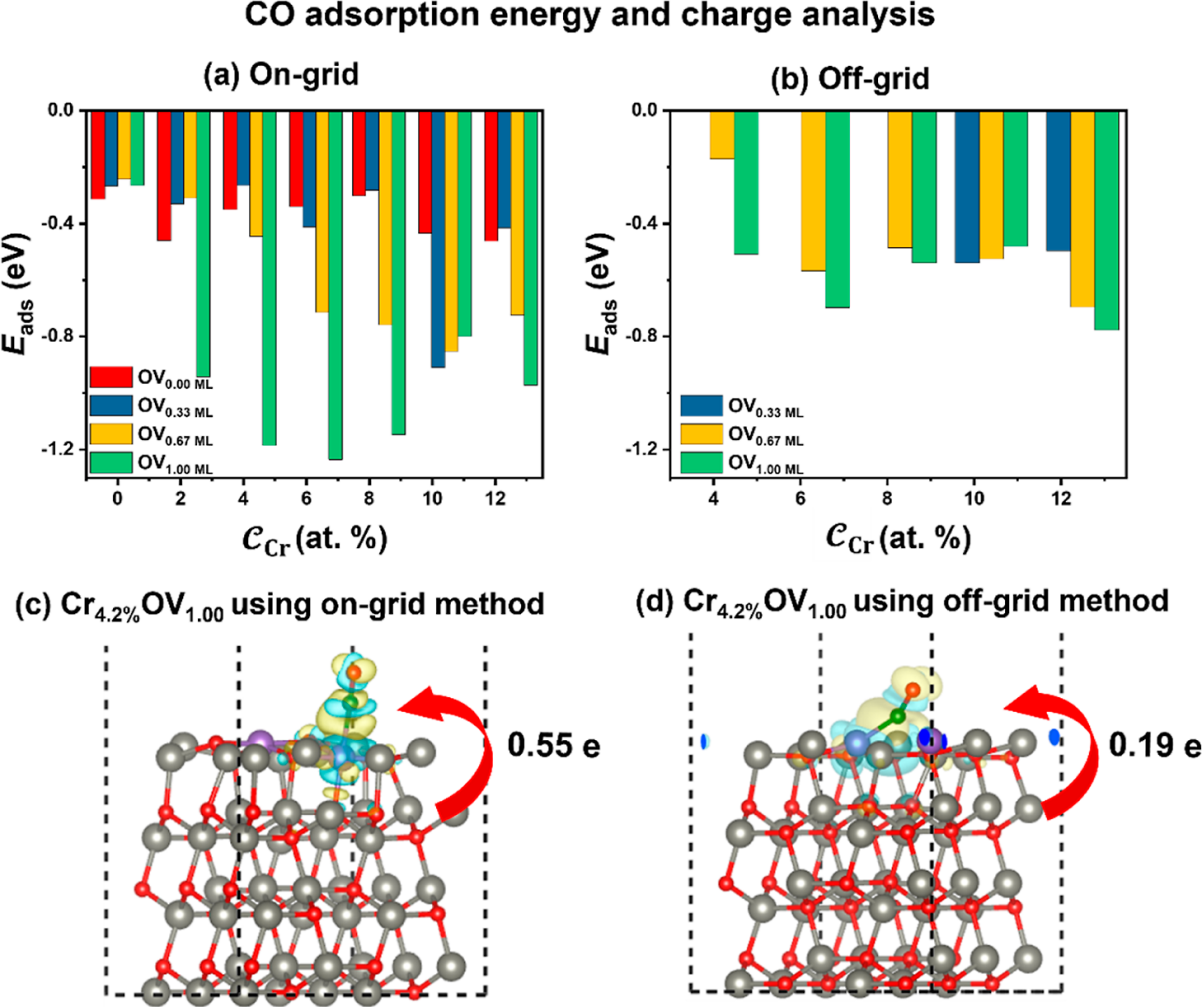
Adsorption energies of CO on structures identified using the on-grid strategy (a) and the off-grid strategy (b), respectively. (c,d) Charge density differences induced by CO adsorption in ZnO with Cr_4.2%_OV_1.00_ identified by the two methods at an isosurface value of 1 × 10^–3^ e Å^–3^. Yellow and blue bubbles represent charge accumulation and depletion, respectively. Color code: gray, red, purple, and green represent Zn, O, Cr, and C, respectively.

After identifying the optimal  of the Cr-doped ZnO surfaces under the reaction conditions, a systematic investigation of CO activation on Cr-doped ZnO surfaces with various  and  were conducted. The CO adsorptions on the Cr-doped ZnO surfaces were calculated at  of 0.00, 0.33, 0.67, and 1.00 ML. The results of the CO adsorptions on the unreconstructed surfaces are shown in Figure 7a. On the surface of ZnO without Cr doping, the presence of OV has little effect on the adsorption of CO (−0.31 to −0.46 eV). Similarly, the effect of Cr doping on the CO adsorption is also very small (−0.24 to −0.31 eV) without any OVs. Experimentally, no preferred adsorption of CO on the lattice Cr sites was observed at 78 K.^18^ However, when  is large enough that the oxygen attached to Cr leaves in the presence of Cr, the CO adsorption energy can be sufficiently strengthened (around −1.0 eV). The CO adsorptions on the reconstructed surfaces were also studied (Figure 7b). The increase magnitude of the adsorption energy with the increase of  and  is relatively small compared to the corresponding unreconstructed structures. Interestingly, we find that the CO-induced stabilization effect for on-grid structures outweighs the energetic favorability of the off-grid-derived surface, leading to a reversal in the relative stability of the two systems (see Tables S3 and S4). Then we investigated the geometries and charge transfers of CO adsorption on the reconstructed and unreconstructed surfaces using Cr_2.1%_OV_1.00_ as an example. The two-coordinated Cr sites of the unreconstructed surface can enhance the charge transfer more than that of the 4-fold-coordinated Cr sites of the reconstructed surface, significantly strengthening the adsorption of CO (see Figure 7c,d). It is clear that the CO adsorption can be substantially enhanced when the Cr and OV coexist. The most stable adsorption configuration can be seen on the two-coordinated Cr sites of the unreconstructed surface.

**Figure 8:**
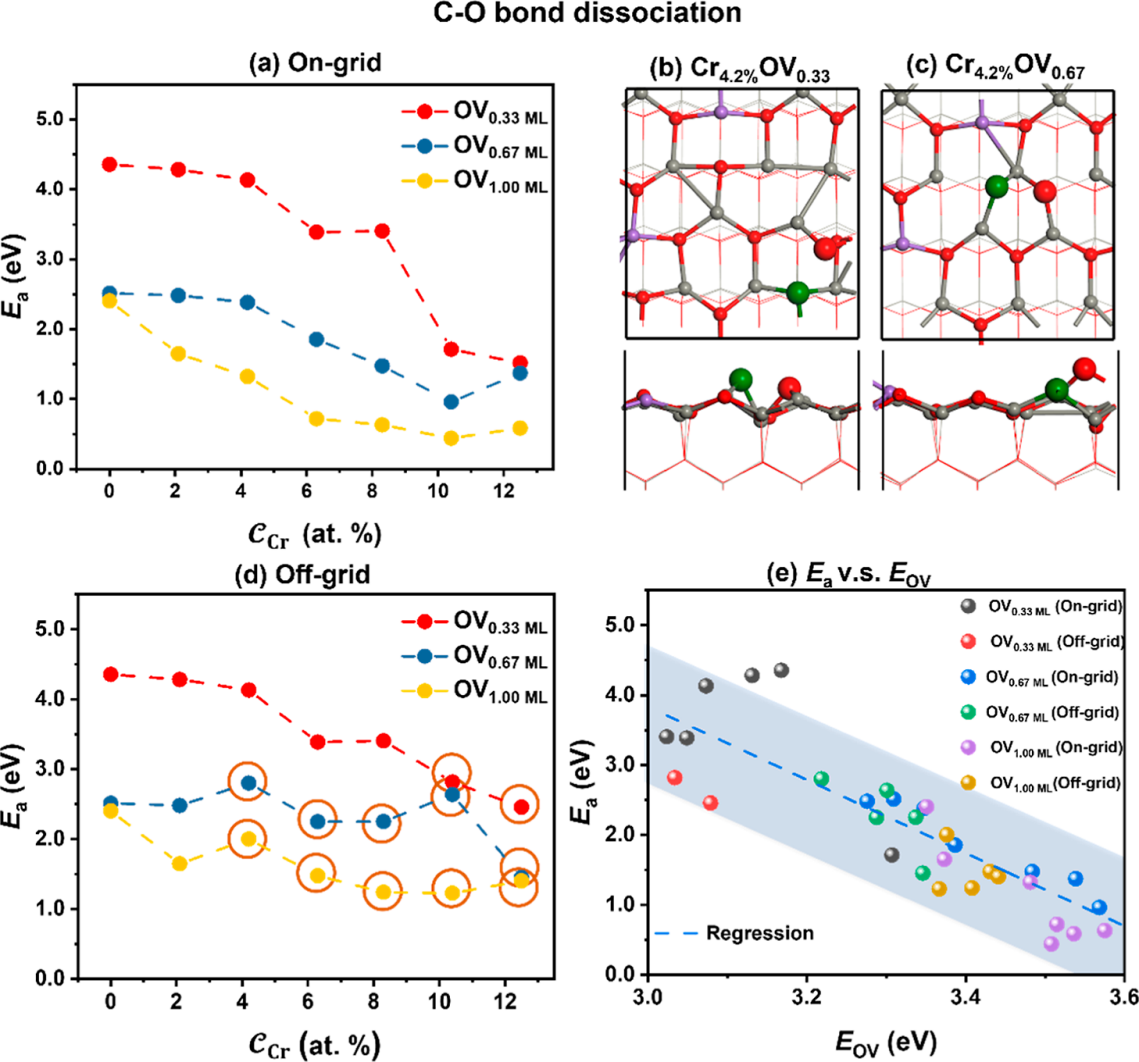
(a) Changes of *E*_a_ on the structures with the increasing  and  based on the on-grid strategy. (b,c) The optimized TSs of C–O bond dissociation on the ZnO surfaces with Cr_4.2%_OV_0.33_ and Cr_4.2%_OV_0.67_. (d) Changes of *E*_a_ on the structures with the increasing  and  based on the off-grid strategy. The red circles represent different structures for the same component found by the two methods. (e) Plots of *E*_a_ against *E*_OV_.

We also conducted a quantitative investigation of the impact of  and  on the cleavage of C–O bonds on the Cr-doped ZnO surfaces. First, we calculated the CO dissociation on the unreconstructed surfaces (see Figure 8a). *E*_a_ decreases significantly with increasing , particularly from OV_0.33 ML_ to OV_0.67 ML_ with a drop of *E*_a_ of nearly 2.0 eV. This configuration effect is ascribed to the geometry of the reaction site (see Figure 8b,c, for further insights and a more detailed discussion, see Section 3.2.3). Meanwhile, with the increased  to 12.5%, *E*_a_ decreases gradually by 1.1–2.8 eV. It is found that with a  of 4.2%, *E*_a_ is decreased significantly when  is varied from 8.3 to 10.4% because one of the cleavage products can be stabilized by the two adjacent Cr ions on the surfaces, as shown in Figure S12. An increase in  from 10.4 to 12.5% results in a slight increase of *E*_a_ rather than a decrease. This is because the deformed Cr-doped ZnO structure is more stable, which is not conducive to the activation of the C–O bonds.

Next, we studied the activation of C–O bonds on the reconstructed surfaces (Figure 8d). It is found that with the increases of  and , the trend of the C–O bond dissociation barriers was almost the same as that of the unreconstructed surfaces: As  increases, *E*_a_ of the C–O bond breaking decreases by 1.0–1.9 eV. As shown in Figure S13, the reconstructed surfaces of the same composition display varying degrees of increases (0.1–1.7 eV) in *E*_a_ compared to the unconstructed surfaces, due to the increased stabilities of the surfaces and the increases of Cr coordination numbers after the structure reconstruction.

As mentioned above, we observed that  notably influences the formation of OVs and both contribute to *E*_a_. Therefore, it is worth studying the relationship between *E*_a_ and *E*_OV_ to further understand the catalytic reaction. A nearly linear relationship between *E*_OV_ and *E*_a_ for CO activation on the structures examined above is found (see Figure 8e). It is noted that *E*_a_ does not fit into a perfect linear relationship with *E*_OV_ (see more discussions in Section 3 of the Supporting Information and Figure S14). The negative slope indicates that the larger the *E*_OV_ is, the lower the *E*_a_ is for the C–O bond activation. In the literature, the Brønsted–Evans–Polanyi (BEP) relationship, namely, the dissociation *E*_a_ is a linear function of the total chemisorption energy of dissociation products, is well-known. In this work, a more simplified model that connects the dissociation barrier *E*_a_ to a simple property, *E*_OV_, was identified. This relationship may provide a way to deduce energy barriers without the time-consuming calculation of TSs.

#### 3.2.3 Impact of Cr and OV Distribution on the CO Activation over Cr-Doped ZnO(1010) Surfaces

**Figure 9:**
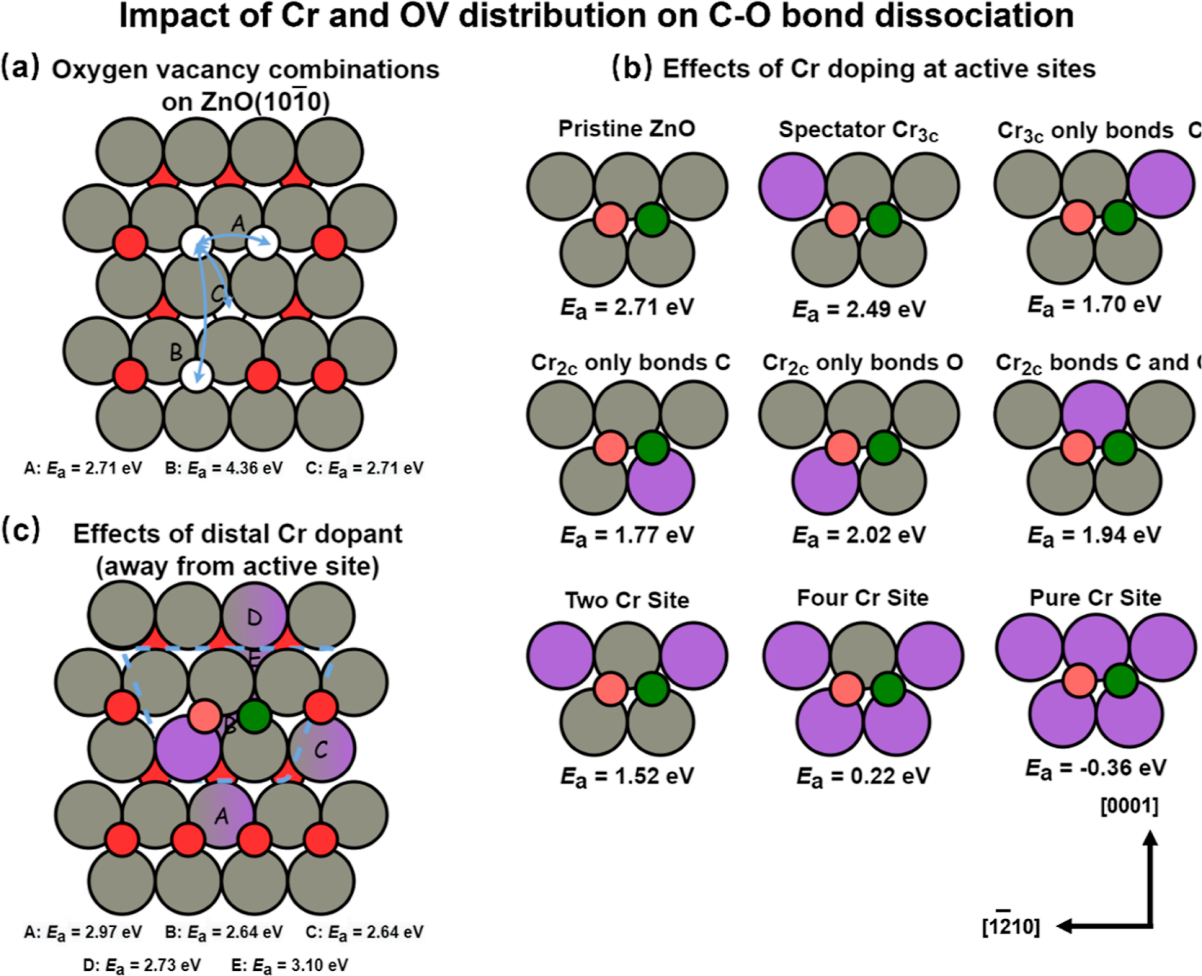
(a) Effects of different arrangements of OVs on *E*_a_. (A, B) represent two OVs are clustered in the [1210] direction and [0001] direction, respectively. (C) illustrates the configuration with a connection between a 3-coordinated surface OV and a 4-fold-coordinated subsurface OV (see Figure S15). (b) Effects of Cr doping at the active sites on *E*_a_. (c) Effects of the distal Cr dopant (away from the reaction site) on *E*_a_. Color code: gray, purple, red, green, and pink represent Zn, Cr, and O of the metal oxide and C and O of CO, respectively.

In this section, we discuss the impact of Cr and OV distributions on the activation of the C–O bond. Under the given reaction condition (Δμ_O_ = −3.3 eV), it is observed that the surface with 0.33 ML OVs tends to exhibit the highest stability across a wide range of . Additionally, as reported above, the unreconstructed Cr-doped ZnO surface exhibits higher activity compared with the reconstructed surface. Consequently, we conducted a detailed analysis using the 0.33 ML OVs (2OVs) model and employed a controlled variable method to examine how the distribution of OV and Cr influences the activity of the unreconstructed ZnO surface. It is observed that the activity can be significantly enhanced by 1.65 eV when two OVs are clustered in the [1210] direction or connected by a 3-coordinated surface OV and a 4-fold-coordinated subsurface OV (see Figure 9a). As shown in Figure S16, when the two OVs are arranged along the direction of [0001], CO can be cracked at only a single OV site; one of the dissociation products fills the empty OV, and the other combines with the two metal sites on the surface. In this way, the activation energy *E*_a_ is relatively high. However, when the OVs are connected geometrically, CO can be cleaved at the two adjacent OVs, whereby the products can be packed into OVs separately. In this way, the TS is more stable, and hence *E*_a_ is greatly reduced. This also explains why the OV_0.67 ML_ structure, where geometrically connected OVs can be observed, decreases the C–O bond activation barrier significantly compared to OV_0.33 ML_, where the 2OVs are arranged along the [0001] direction (see Figure 8b,c). These findings highlight the crucial role played by the arrangement of OVs in the C–O bond activation process.

On the other hand, oxygen located on the subsurface (C arrangement) is difficult to remove with a higher *E*_OV_ by 0.39 eV compared to the [1210] direction arrangement (A arrangement). Then we chose the local environment when the 2OVs are aligned along the [1210] direction as the active center to further study the effect of Cr doping at or away from the active center on the activity, respectively. When Cr acts as the active center, there is a significant increase in the activity (see Figure 9b). If Cr is connected to C, then *E*_a_ can be considerably reduced. As  increases, *E*_a_ decreases significantly, even reaching −0.36 eV (the effective barrier with respect to CO in the gas phase) when Cr replaces all the metals in the active center. However, as Cr was placed away from the active sites, *E*_a_ cannot be effectively reduced (see Figure 9c). These results clearly demonstrate that the local environment with OVs connected geometrically and Cr doped in the active sites can effectively reduce the *E*_a_ of C–O bond activation.

### 3.3 Discussion on the On-Grid and Off-Grid Methods for Exploring Reduced Oxide Surfaces

Through the investigation of Zn–Cr–O surfaces using both on-grid and off-grid strategies, complemented by the calculations of C–O bond dissociation on these surfaces, the following two key features worth discussing have emerged. First, it is commonly observed that calculations of the OV formation primarily employed the on-grid strategy, which usually maintains the crystal lattice structure. However, our findings challenge the thermodynamic stability of such unreconstructed structures. With the increase of , Cr ions tend to agglomerate together to form a stripe structure along the [0001] direction. We suggest that the global optimization methods that encompass a broader range of structural possibilities may be more adopted to gain deeper insights into real systems. Second, both the unreconstructed structures and reconstructed ones may be observable under the reaction conditions. The unreconstructed structures, which are obtained by local optimizations (the on-grid optimization in this work), may exist shortly after they are produced under real experimental conditions. For example, a surface on which some OVs are just generated may not be allowed to have enough time to have quite complicated reconstruction before some surface reactions take place on the surface. Conversely, if a surface in the presence of OVs is left for an extended period without surface reactions, the surface may gradually undergo a reconstruction process, resulting in a reconstructed structure, which is obtained by global structural optimizations (the off-grid structural optimization in this work). Based on our computational results, the reconstructed surface is found to exhibit higher stability; however, it is accompanied by a reduction in both active sites and activity. This significant observation offers significant insights into catalyst design, underscoring the critical role of experimentally obtaining these structures to effectively optimize their performance.

## 4 Conclusions

This work employed ML-based GCMC and GA global exploration methods to identify the reduced surfaces of Cr-doped ZnO(1010) under the reactive condition and quantitatively studied the effects of  and  on the activation of CO molecules. The main conclusions are summarized as follows:

(1) Cr ions have a greater affinity to move toward the surface of ZnO(1010) than to remain in its interior.
(2) OVs can induce significant structural changes in the system: Cr-doped ZnO surfaces are prone to be reconstructed, resulting in the formation of islands along the [0001] direction with the increasing  and . As  increases, the  decreases under equilibrium conditions. After surface reconstruction, the reduction degree of ZnO surfaces is observed to be greater.
(3) On the Cr-doped ZnO surfaces, the removal of the O attached to Cr significantly enhances CO adsorption. The two-coordinated Cr site in the unreconstructed surface exhibits a stronger CO adsorption energy than the 4-fold-coordinated Cr site on the reconstructed surface for the same composition.
(4) Under the reaction conditions, the ZnO surface with 10.4%  shows the highest activity toward C–O bond dissociation. With the increased  and , the C–O dissociation barrier *E*_a_ decreases. The reconstructed configuration exhibits relatively poor activity due to the stability of the reconstructed structure. A nearly linear relationship between *E*_a_ and *E*_OV_ was identified. It may be possible to estimate activation energies for C–O activation solely from information on the OV formation energy.
(5) By studying the effect of Cr and OV distribution on the C–O bond dissociation, we found that the activity can be significantly increased when the OVs are aligned along the [1210] direction and doped with Cr in the active center, which is crucial information for obtaining high activity of metal oxide catalysts for CO activation.

Through structure determinations and activity calculations using a set of the-state-the-art methods, this work strengthens our understanding of the activation of C–O bonds on Cr-doped ZnO surfaces and provides new insights into the rational design of catalysts toward syngas conversion. The principles explored here can be extended to the study of the general structures of metal-doped oxide surfaces, providing valuable insights for future research in this area.

## Supporting Information Available

Details of the sensitive analysis of U values, the GA global optimization accelerated by the MLPs and oxygen chemical potential (μ_O_) calculation; full list of data sets; DOSs of the optimized structures; the optimized TSs of the C–O bond dissociation (PDF)
DFT-optimized structures of the most stable surfaces identified using on-grid and off -grid methods (ZIP)
