# Supplementary material for: Unravelling the Impact of Metal Dopants and Oxygen Vacancies on Syngas Conversion over Oxides: A Machine Learning-Accelerated Study of CO Activation on Cr-Doped ZnO Surfaces
Source: ACS Catal. 2023 Nov 8;13(22):15074–86. doi: 10.1021/acscatal.3c03648 (PMC10660660; doi:10.1021/acscatal.3c03648)
Supplement: Supplementary file 1 — cs3c03648_si_001.pdf [file cs3c03648_si_001.pdf]

Supporting Information

Unravelling the Impact of Metal Dopants and Oxygen Vacancies on Syngas Conversion over Oxides: A Machine Learning-Accelerated Study of CO Activation on Cr-Doped ZnO Surfaces

Yulan Han[1,2#], Jiayan Xu[1#], Wenbo Xie[1,2], Zhuozheng Wang[1,3], P. Hu[1,2*]

[1]*School of Chemistry and Chemical Engineering, Queen's University Belfast, Belfast BT9 5AG, UK*

[2]*School of Physical Science and Technology, ShanghaiTech University, Shanghai 201210, China*

[3]*PetroChina Petrochemical Research Institute, Beijing 102206, China*

Corresponding Author

*E-mail address: p.hu@qub.ac.uk

1. Computational methods

1.1 The sensitive analysis of U values

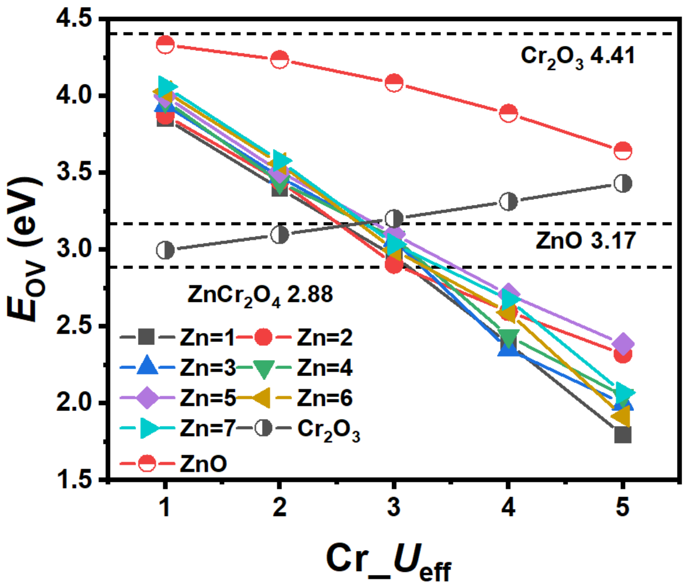

Figure S1. Oxygen vacancy (OV) formation energies (E_{OV}) of ZnO(10 $\bar{1}$ 0), $ZnCr_2O_4$(111) and Cr_2O_3(0001) with different U value in PBE+U calculations. The dash line denotes the E_{OV} calculated based on the HSE calculation.

As shown, the value of U was determined by benchmarking with HSE data and was found to be 3.0 eV for Cr and Zn better, which adequately described the E_{OV} for various systems containing Zn or Cr. E_{OV} was selected for benchmarking due to its significance in evaluating the equilibrium OV concentrations under the reaction condition, which has a great impact on the C-O bond activation.

1.2 ML-accelerated global optimization methods

Table S1. Energy and force root mean squared error (RMSE) on DFT-labelled structures. The Nframes is the number of the training structures, as distinguished by the chemical formula (system) and the number of atoms per cell (Natoms).

System	Natoms	Nframes	E_{RMSE} (eV/atom)	F^{Cr}_{RMSE} (eV/Å)	F^{o}_{RMSE} (eV/Å)	F^{Zn}_{RMSE} (eV/Å)
Cr0O0Zn14	14	25	0.0157	nan	nan	0.0368
Cr0O0Zn15	15	683	0.0161	nan	nan	0.0791
Cr0O0Zn16	16	4164	0.0126	nan	nan	0.0815
Cr0O0Zn17	17	8	0.0107	nan	nan	0.0431
Cr0O0Zn18	18	160	0.0034	nan	nan	0.0492
Cr0O0Zn19	19	63	0.0153	nan	nan	0.0819
Cr0O0Zn22	22	80	0.0077	nan	nan	0.083

Cr0O0Zn24	24	229	0.0143	nan	nan	0.0519
Cr0O0Zn28	28	22	0.0123	nan	nan	0.0412
Cr0O0Zn29	29	18	0.0211	nan	nan	0.1111
Cr0O0Zn30	30	56	0.046	nan	nan	0.2016
Cr0O0Zn31	31	67	0.0091	nan	nan	0.0535
Cr0O0Zn32	32	87	0.0122	nan	nan	0.0415
Cr0O10Zn10	20	1411	0.0084	nan	0.0794	0.0734
Cr0O10Zn16	26	75	0.0111	nan	0.1344	0.139
Cr0O10Zn24	34	334	0.008	nan	0.1381	0.1183
Cr0O11Zn0	11	790	0.0337	nan	0.4732	nan
Cr0O11Zn15	26	28	0.0135	nan	0.1496	0.1524
Cr0O11Zn16	27	33	0.0093	nan	0.1595	0.1795
Cr0O12Zn16	28	2916	0.0264	nan	0.16	0.1542
Cr0O13Zn15	28	231	0.0098	nan	0.1137	0.1192
Cr0O13Zn16	29	2727	0.0264	nan	0.3116	0.3272
Cr0O14Zn15	29	53	0.0132	nan	0.1179	0.1175
Cr0O14Zn16	30	206	0.0093	nan	0.1144	0.117
Cr0O15Zn16	31	3133	0.0539	nan	0.1791	0.1469
Cr0O16Zn14	30	341	0.092	nan	0.2066	0.1574
Cr0O16Zn16	32	445	0.0058	nan	0.09	0.0878
Cr0O2Zn17	19	250	0.0106	nan	0.145	0.0909
Cr0O36Zn48	84	473	0.0024	nan	0.044	0.0668
Cr0O37Zn48	85	294	0.0121	nan	0.0555	0.0807
Cr0O38Zn48	86	475	0.0035	nan	0.0646	0.0864
Cr0O39Zn48	87	492	0.0037	nan	0.0807	0.094
Cr0O40Zn48	88	491	0.0122	nan	0.1057	0.1017
Cr0O41Zn48	89	413	0.0129	nan	0.1301	0.1135
Cr0O42Zn48	90	454	0.0041	nan	0.1034	0.1081
Cr0O43Zn48	91	493	0.0126	nan	0.1274	0.1171
Cr0O44Zn48	92	521	0.0346	nan	0.3235	0.1282
Cr0O45Zn48	93	508	0.016	nan	0.1265	0.1263
Cr0O46Zn48	94	465	0.0052	nan	0.1218	0.1095
Cr0O47Zn48	95	521	0.0291	nan	0.1716	0.1271
Cr0O48Zn48	96	2041	0.0032	nan	0.0767	0.071
Cr0O4Zn0	4	79	0.0587	nan	0.2275	nan
Cr0O4Zn15	19	26	0.012	nan	0.1302	0.1039
Cr0O6Zn4	10	40	0.0584	nan	0.8937	0.5478
Cr0O6Zn6	12	1415	0.0176	nan	0.1678	0.1606
Cr0O6Zn8	14	306	0.0218	nan	0.1512	0.1481
Cr0O7Zn16	23	33	0.0113	nan	0.1393	0.1296
Cr0O7Zn8	15	309	0.0204	nan	0.1443	0.1506
Cr0O8Zn16	24	1129	0.0046	nan	0.0478	0.0765
Cr0O8Zn8	16	3588	0.0092	nan	0.0752	0.063
Cr0O9Zn16	25	1295	0.0066	nan	0.088	0.1003

Cr10O14Zn2	26	81	0.0136	0.2001	0.1879	0.1404
Cr10O18Zn2	30	62	0.0124	0.2605	0.2183	0.1352
Cr10O20Zn8	38	160	0.0367	0.1965	0.1482	0.1329
Cr10O22Zn8	40	160	0.0451	0.2059	0.1653	0.1779
Cr10O23Zn7	40	31	0.0064	0.2457	0.1819	0.1465
Cr10O24Zn7	41	44	0.0109	0.2645	0.203	0.1333
Cr10O24Zn8	42	160	0.025	0.2167	0.1916	0.1169
Cr11O23Zn6	40	40	0.0126	0.2236	0.2002	0.1277
Cr11O24Zn6	41	37	0.0072	0.2262	0.1792	0.1124
Cr12O14Zn0	26	47	0.0185	0.1904	0.1925	nan
Cr12O16Zn0	28	132	0.0192	0.1073	0.1054	nan
Cr12O16Zn0	28	651	0.0071	0.1413	0.1337	nan
Cr12O18Zn0	30	2509	0.0019	0.0586	0.0474	nan
Cr15O39Zn15	69	40	0.0078	0.2619	0.2049	0.137
Cr15O40Zn15	70	53	0.0068	0.2768	0.2031	0.1386
Cr16O31Zn8	55	88	0.0111	0.2515	0.1946	0.1408
Cr16O32Zn0	48	14	0.0359	1.0694	1.1065	nan
Cr16O32Zn1	49	232	0.0083	0.265	0.2072	0.142
Cr16O32Zn4	52	12	0.0275	1.1195	1.0513	0.1423
Cr16O32Zn5	53	26	0.0217	0.4682	0.5302	0.1548
Cr16O32Zn8	56	106	0.0109	0.2424	0.1957	0.1385
Cr16O32Zn8	56	746	0.0176	0.16955	0.12885	0.08125
Cr1O7Zn5	13	77	0.0233	0.359	0.2011	0.1356
Cr1O8Zn5	14	514	0.0235	0.3146	0.1951	0.1518
Cr24O47Zn12	83	34	0.0107	0.2533	0.1995	0.1394
Cr24O48Zn12	84	38	0.0124	0.2378	0.1863	0.1314
Cr29O64Zn19	112	160	0.0046	0.1841	0.152	0.1209
Cr2O16Zn10	28	139	0.0111	0.2579	0.169	0.1642
Cr2O42Zn46	90	5897	0.0158	0.1974	0.0788	0.0806
Cr2O43Zn46	91	2781	0.0065	0.2026	0.0841	0.0828
Cr2O44Zn46	92	4173	0.0036	0.2182	0.0882	0.0831
Cr2O45Zn46	93	4220	0.0034	0.2164	0.0845	0.076
Cr2O46Zn46	94	4931	0.0031	0.2149	0.0846	0.0721
Cr2O47Zn46	95	11034	0.003	0.2241	0.0875	0.0687
Cr2O6Zn4	12	189	0.0156	0.171	0.1383	0.1168
Cr2O7Zn4	13	85	0.0127	0.2062	0.16	0.1192
Cr2O8Zn4	14	109	0.0326	0.345	0.254	0.1564
Cr30O64Zn18	112	160	0.0054	0.1992	0.2054	0.1294
Cr31O64Zn17	112	127	0.0065	0.2046	0.1657	0.1158
Cr32O60Zn16	108	160	0.0056	0.1878	0.1507	0.1062
Cr32O61Zn16	109	160	0.016	0.1951	0.1667	0.1144
Cr32O62Zn16	110	160	0.0376	0.2088	0.2402	0.1248
Cr32O63Zn16	111	92	0.0045	0.1895	0.156	0.1072
Cr3O16Zn9	28	10	0.0282	0.3823	0.2656	0.1711

Cr3O6Zn3	12	246	0.0146	0.1622	0.157	0.1394
Cr3O8Zn3	14	166	0.0177	0.23	0.1885	0.1405
Cr4O12Zn8	24	101	0.011	0.1389	0.12	0.0939
Cr4O6Zn0	10	309	0.0246	0.1938	0.1657	nan
Cr4O6Zn2	12	2560	0.0231	0.2063	0.1913	0.1357
Cr4O7Zn2	13	7232	0.032	0.3008	0.222	0.1494
Cr4O8Zn0	12	180	0.0261	0.2632	0.2118	nan
Cr4O8Zn1	13	33	0.0226	0.3346	0.2908	0.1447
Cr4O8Zn2	14	8604	0.0286	0.2624	0.1896	0.1292
Cr4O8Zn3	15	37	0.0194	0.1969	0.196	0.1186
Cr5O16Zn6	27	4	0.0086	0.298	0.2827	0.1519
Cr5O16Zn7	28	24	0.0175	0.2802	0.2209	0.1306
Cr5O6Zn1	12	58	0.0111	0.1102	0.1149	0.0679
Cr5O7Zn1	13	243	0.0197	0.2337	0.2075	0.1415
Cr5O8Zn1	14	2384	0.0277	0.2875	0.2283	0.1463
Cr6O12Zn6	24	90	0.0108	0.1747	0.1576	0.1537
Cr6O14Zn6	26	31	0.0121	0.2351	0.1939	0.1197
Cr6O15Zn5	26	18	0.0128	0.2628	0.2151	0.1327
Cr6O16Zn4	26	205	0.0102	0.2887	0.1993	0.1023
Cr6O16Zn6	28	23	0.0092	0.2562	0.2228	0.1299
Cr6O18Zn6	30	137	0.009	0.3307	0.2201	0.1261
Cr6O7Zn0	13	274	0.0255	0.198	0.1861	nan
Cr6O8Zn0	14	2404	0.0307	0.2287	0.1937	nan
Cr7O14Zn4	25	16	0.0144	0.2093	0.1795	0.1328
Cr7O20Zn11	38	160	0.0091	0.1317	0.1248	0.1331
Cr7O22Zn11	40	160	0.0317	0.1594	0.1574	0.1407
Cr7O24Zn11	42	160	0.0486	0.1599	0.1827	0.1617
Cr8O12Zn0	20	129	0.0271	0.2259	0.1781	nan
Cr8O14Zn4	26	61	0.0149	0.2051	0.1948	0.1268
Cr8O16Zn0	24	467	0.0088	0.199	0.1503	nan
Cr8O16Zn4	28	51	0.0118	0.2035	0.1614	0.1125
Cr8O18Zn4	30	41	0.0136	0.2755	0.2119	0.1426
Cr8O19Zn5	32	94	0.0103	0.3772	0.2306	0.4104
Cr8O20Zn10	38	160	0.0407	0.1626	0.1411	0.143
Cr8O22Zn10	40	160	0.0089	0.1398	0.1185	0.1079
Cr8O24Zn10	42	160	0.0277	0.1903	0.173	0.1377
Cr8O8Zn0	16	700	0.0028	0.0643	0.0603	nan
Cr9O16Zn3	28	13	0.0124	0.2372	0.2119	0.1309
Cr9O20Zn9	38	160	0.0108	0.1699	0.1353	0.1305
Cr9O21Zn8	38	56	0.0113	0.2676	0.2069	0.1482
Cr9O22Zn5	36	73	0.0134	0.2954	0.2272	0.1773
Cr9O22Zn6	37	200	0.0134	0.3195	0.2535	0.1817
Cr9O22Zn7	38	207	0.0116	0.28	0.2281	0.2072
Cr9O22Zn8	39	132	0.0082	0.2508	0.2182	0.1848

Cr9O22Zn9	40	160	0.0117	0.1755	0.1466	0.1162
Cr9O23Zn8	40	48	0.0109	0.234	0.1913	0.1317
Cr9O23Zn9	41	115	0.009	0.2489	0.199	0.1424
Cr9O24Zn8	41	47	0.0074	0.2824	0.2325	0.1654
Cr9O24Zn9	42	2845	0.0111	0.2338	0.1937	0.1357
Total	0	107069	0.0209	0.2227	0.1299	0.0993

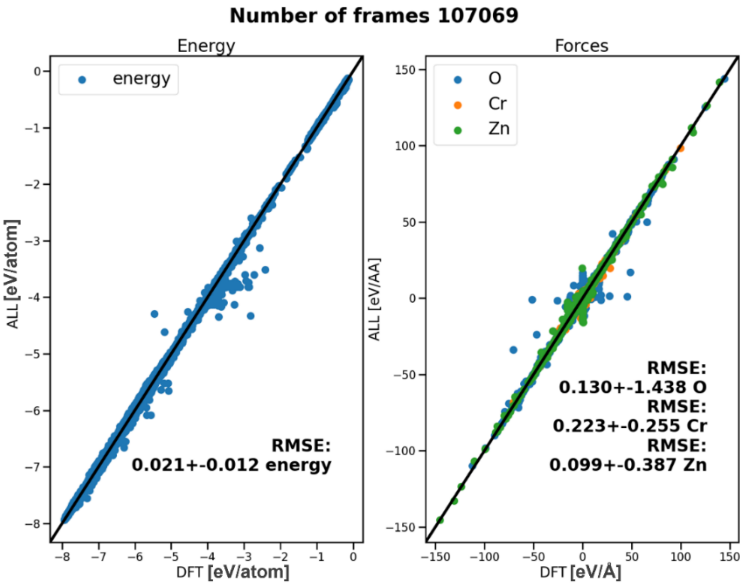

Figure S2. Energy and force root mean squared error (RMSE) on DFT-labelled structures.

1.3 Oxygen chemical potential (μ_O) calculation

We defined the chemical potential of the last OV removed as μ_{O}, and $\mu_{\mathrm{O}} = \mu_{\mathrm{CO_2}} - \mu_{\mathrm{CO}}$, considering that the equilibrium conditions are reached by using CO, in which

$$\mu(T,P) = E_{\mathrm{DFT}} + \Delta E_{\mathrm{ZPE}} + U(T) + TS(T) + k_B T ln(\frac{P}{P_0}) \qquad (1)$$

where E_{DFT} can be obtained from the DFT total energy. Vibrational frequency calculations were performed to determine the zero-point energy (ΔE_{ZPE}). $U(T)$ is the enthalpy correction and $S(T)$ is the entropy, taken from the NIST database.[1]

For CO-assisted OV formation, the pressure of CO_2 was evaluated at 673 K, 0.0765 Mpa, according to the experimental results (CO conversation at ~17% and CO_2 selectivity at ~45%).[2] The detailed results are listed in **Table S2**.

Table S2. Correction to gibbs free energies of the molecules.

	E_{DFT}	ZPE	$U(T)$	$TS(T)$	$k_B T ln(\frac{P}{P_0})$	$\mu(T,P)$
CO_2(673K,0.0765 Mpa)	-22.99	0.32	0.28	1.75	-0.02	-24.16
CO(673 K,1 Mpa)	-14.80	0.14	0.21	1.56	0.13	-15.87

H_2O(298 K, 1 atm)	-14.22	0.58	0.06	0.67	0	-14.25
H_2(298 K, 1 atm)	-6.76	0.27	0.09	0.40	0	-6.80

Under the equilibrium condition, the μ_{O} = -8.29 eV. In our work, to see the influence of reducing environment more clearly, we defined $\Delta\mu_{\mathrm{O}} = \mu_{\mathrm{O}} - \tilde{\mu}_{\mathrm{O}}$, where $\tilde{\mu}_{\mathrm{O}}$ is the chemical potential of oxygen under the standard condition (298.15 K, 0.1 Mpa).

$\tilde{\mu}_{\mathrm{O}}$ is computed by the DFT energies of H_2O and H_2. Given that the high-spin ground state of the oxygen molecule is poorly described in DFT calculations, this method to calculate the free energy of the O_2 molecule was derived according to $\mu_{\mathrm{O_2}}(\mathrm{g}) = 2\mu_{\mathrm{H_2O}}(\mathrm{l}) - 2\mu_{\mathrm{H_2}}(\mathrm{g}) + 4 \times 1.23$ (eV) under the standard condition.
In this way, $\tilde{\mu}_{\mathrm{O}}$=1/2 $\tilde{\mu}_{\mathrm{O_2}}$ = -4.99 eV
$\Delta\mu_{\mathrm{O}} = \mu_{\mathrm{O}} - \tilde{\mu}_{\mathrm{O}}$= -8.29 – (-4.99) = -3.30 eV

2. Thermodynamics of the Cr-doped ZnO(10$\bar{1}$0) surface

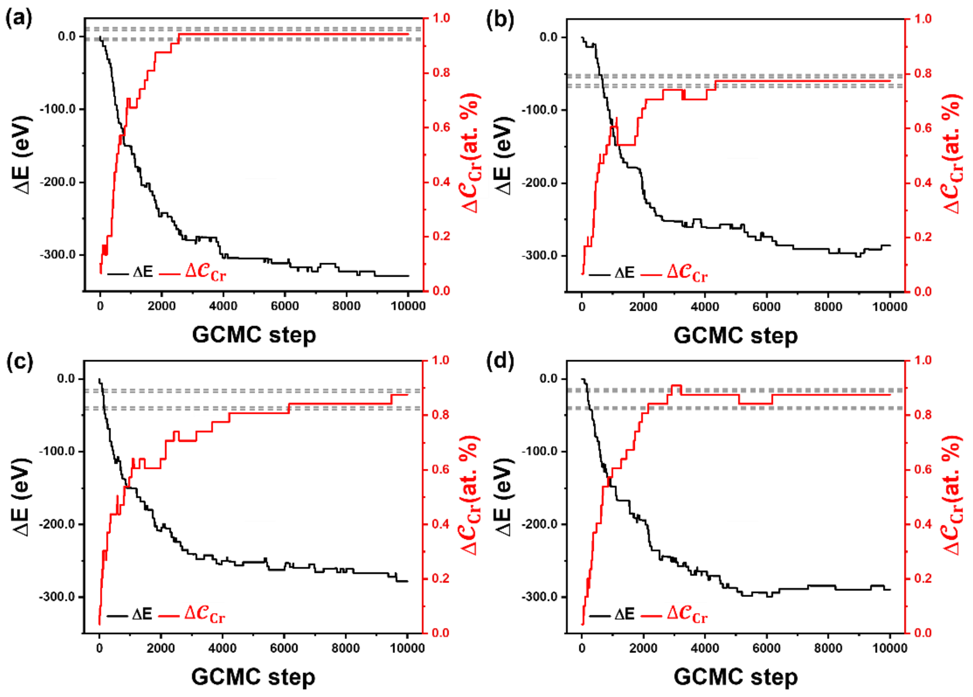

Figure S3. Changes of the total energies (black line) and C_{Cr} (red line) of the surface region(**Figure 2a**) as a function of neural network accelerated GCMC simulation step for ZnO(10$\bar{1}$0) with 1% C_{Cr} at 798 K.

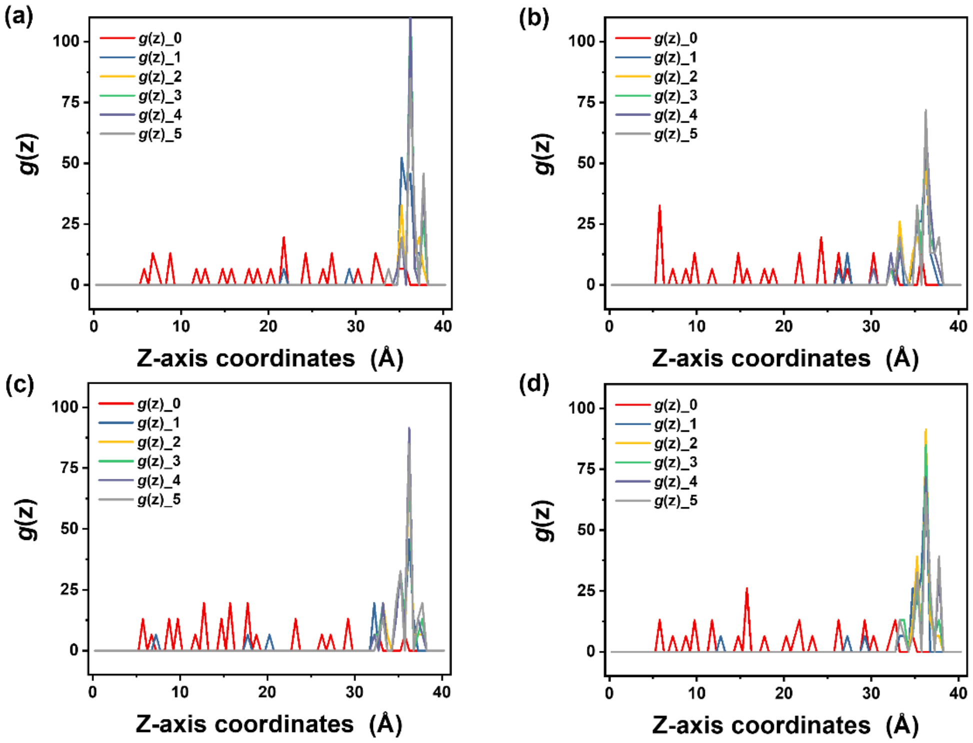

Figure S4. Cr distribution function in z direction ($g(z)$) for the structures obtained from GCMC simulations. $g(z)\_0$ refers to the $g(z)$ in the original structure, and subsequent $g(z)$ analyses were conducted at intervals of 2000 simulation steps.

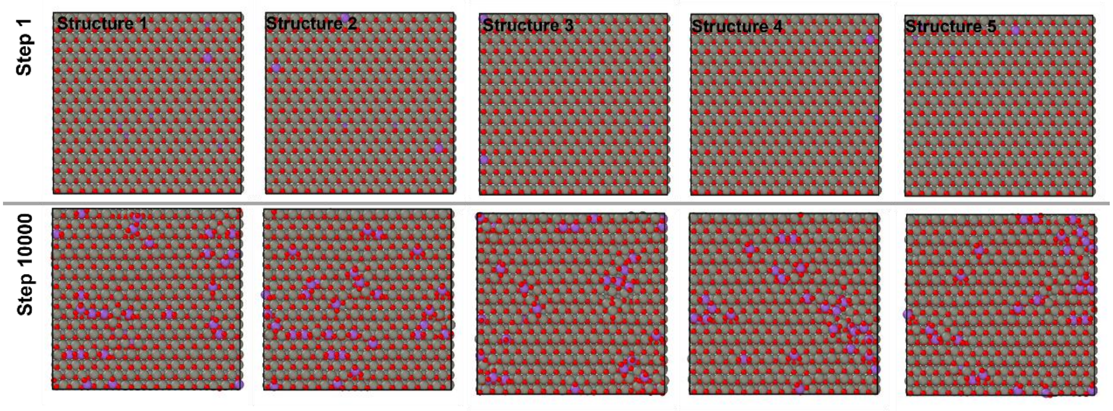

Figure S5. Initial Cr-doped ZnO surfaces and their evolution after 10000 steps of GCMC simulations.[3]

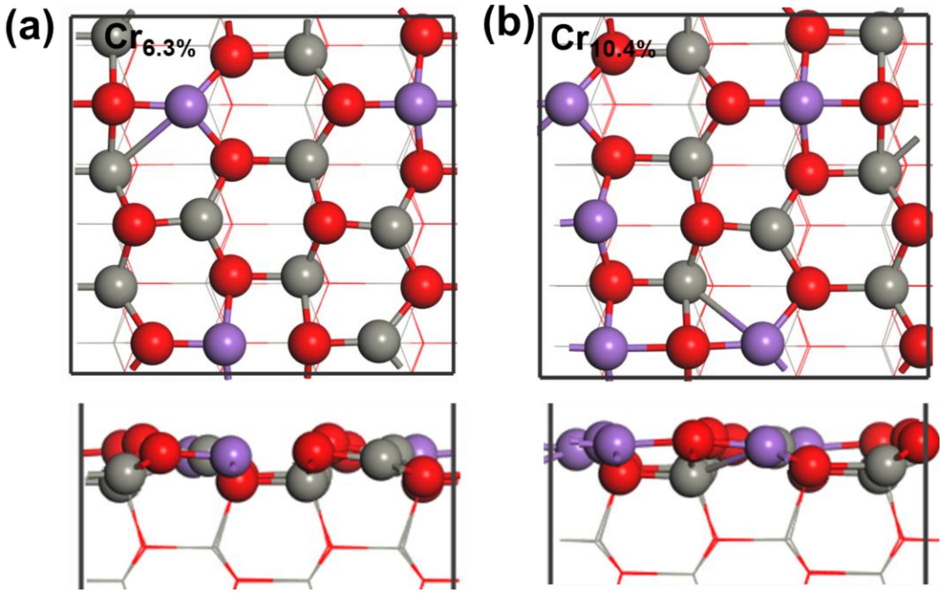

Figure S6. Optimized structures of ZnO surface with (a) 6.3% and (b)10.4% Cr, respectively.

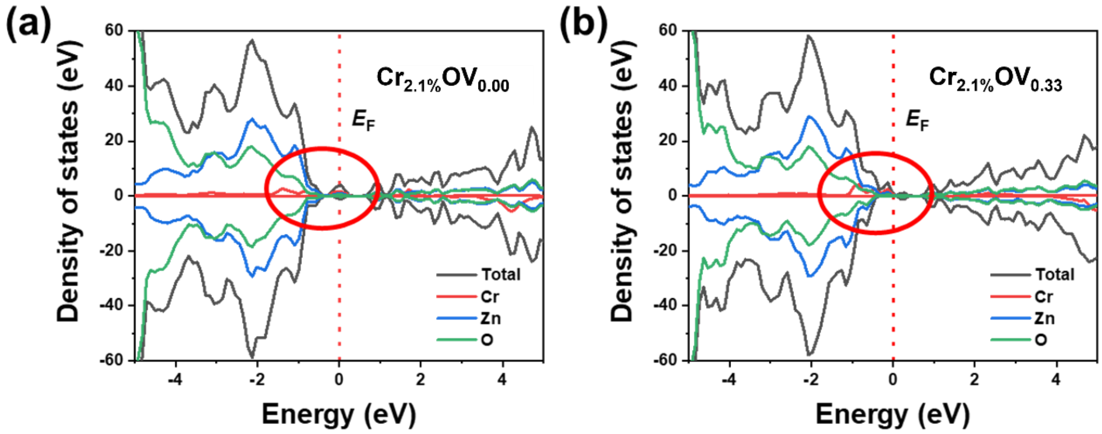

Figure S7. Calculated density of states of ZnO with 2.1% Cr before(a) and after(b) introducing 0.33 ML OVs with respect to the Fermi level.

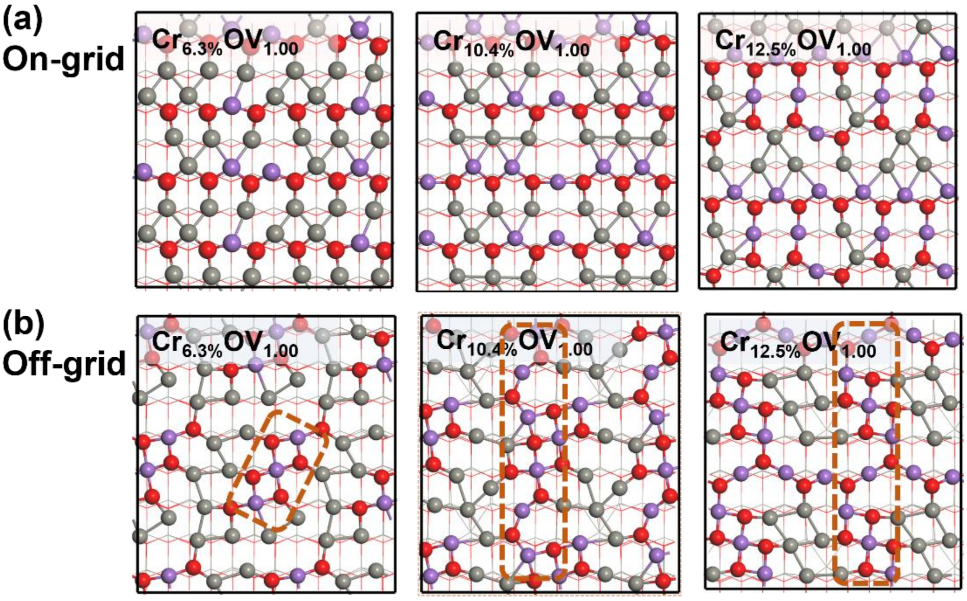

Figure S8. Most stable structures identified by on-grid and off-grid strategies.

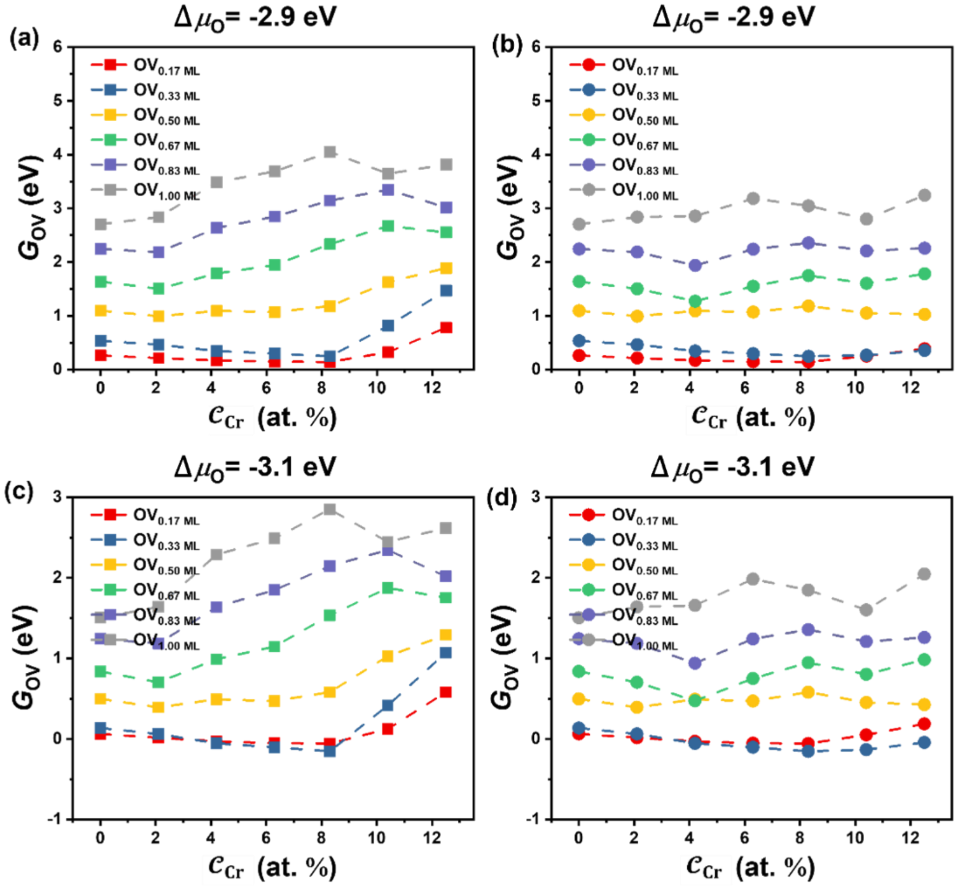

Figure S9. Gibbs free energy of OV formation(G_{OV}) with various $c_{Cr}$and c_{OV} based on the on-grid strategy(a,c) and the off-grid strategy(b,d) under various $\Delta\mu_O$(-2.9, -3.1 eV).

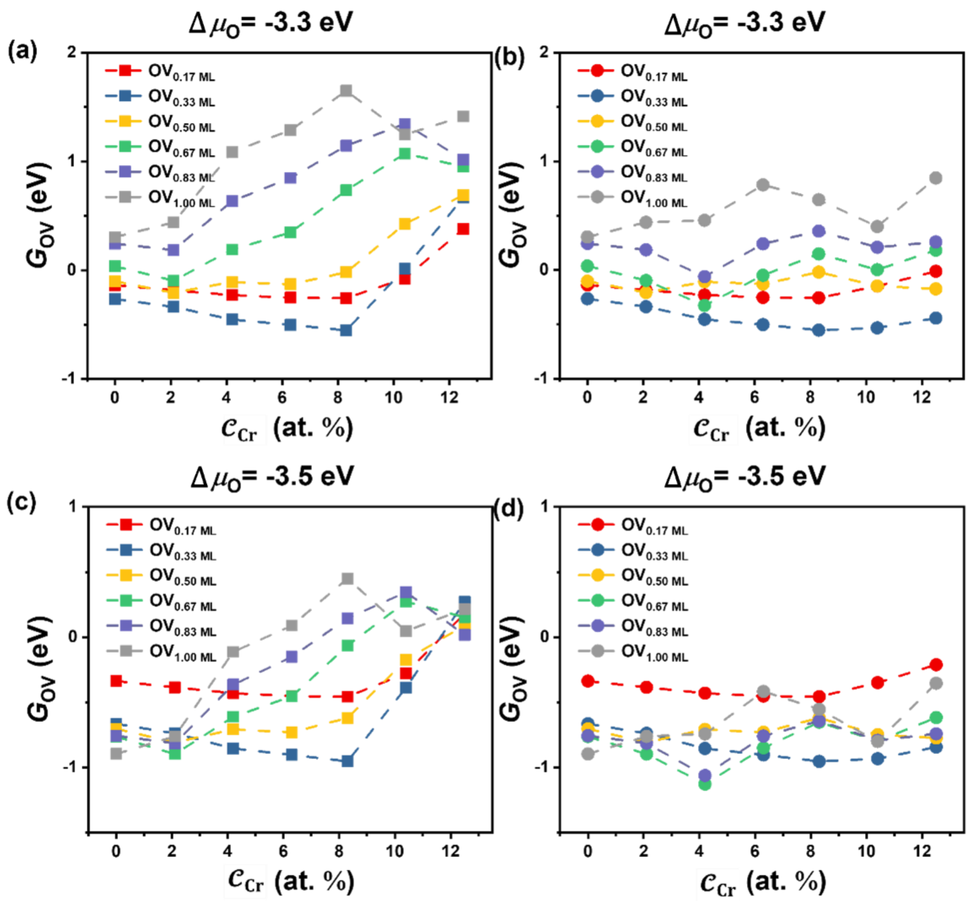

Figure S10. G_{OV} with various $c_{Cr}$and c_{OV} based on the on-grid strategy(a,c) and the off-grid strategy(b,d) under various $\Delta\mu_O$(-3.3, -3.5 eV).

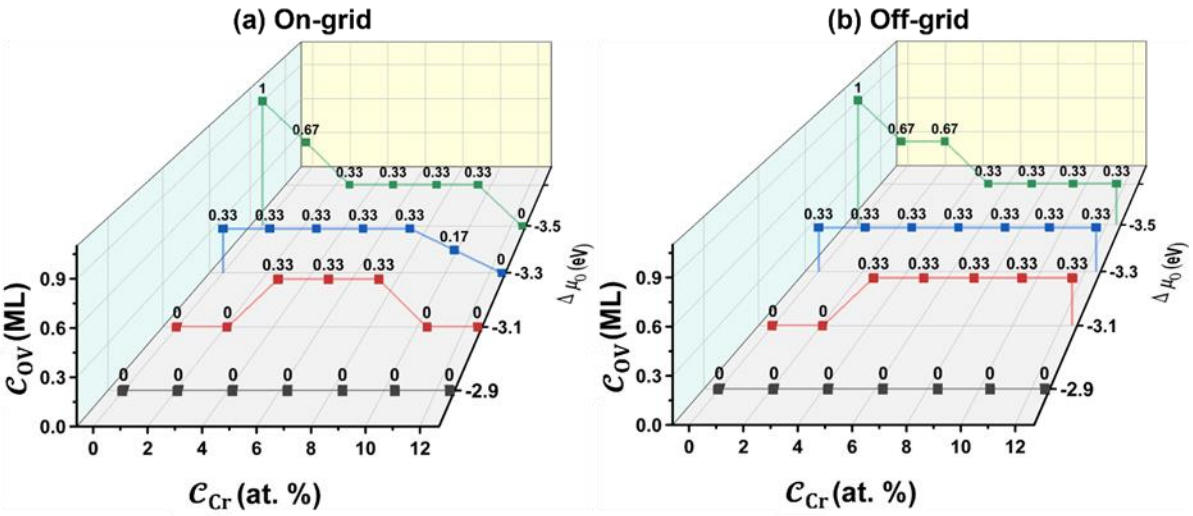

Figure S11. Equilibrium c_{OV} of Cr-doped ZnO identified by on-grid (a) and off-grid (b) methods under different $\Delta\mu_O$.

Under the reaction conditions, these surfaces should undergo reduction by reacting with CO/H_2 to form CO_2/H_2O. The effect of μ_O on the equilibrium c_{OV} was

investigated through the calculation of $G_{\rm OV}$ (**Figures S9** and **S10**). On the unreconstructed surfaces, more OVs can be observed on the surfaces with low $C_{\rm Cr}$ (1.00 ML OVs for 0% $C_{\rm Cr}$ and 0.00 ML OVs for 12.5% $C_{\rm Cr}$ under $\Delta\mu_{\rm O}$ = -3.5 eV) and $C_{\rm OV}$ increases gradually with the decline of $\mu_{\rm O}$, as shown in **Figure S11a**. An intriguing observation is that 0.33 ML OVs can be found when $C_{\rm Cr}$ is between 4.2% to 8.3% under $\Delta\mu_{\rm O}$=-3.1 eV, whereas no OV formation is observed in the lower $C_{\rm OV}$ ranging from 0% to 4.2%. This finding can be attributed to the promotion of OV generation around the Zn ions due to the incorporation of Cr, as mentioned previously. After structural reconstruction, the overall trends remain similar, except that the reduction degree may be greater under the same $\Delta\mu_{\rm O}$ (0.00 and 0.33 ML OVs for 12.5% $C_{\rm Cr}$ under $\Delta\mu_{\rm O}$= -3.3 eV), as can be seen in **Figure S11b**. These results suggest, therefore, that one may manipulate $C_{\rm OV}$ of the surface by adjusting $\mu_{\rm O}$ (temperature and pressure of the experiment).

3. CO activation

Table S3. Energy differences between the most stable structures produced by the off-grid and on-grid methods. The negative sign means that the structure from off-grid is more stable.

$C_{\rm OV}$ \ $C_{\rm cr}$	0.00(ML)	0.33 (ML)	0.67 (ML)	1.00 (ML)
0.0 (at.%)	0.00	0.00	0.00	0.00
2.1 (at.%)	0.00	0.00	0.00	0.00
4.2 (at.%)	0.00	0.00	-0.52	-0.63
6.3 (at.%)	0.00	0.00	-0.40	-0.50
8.3 (at.%)	0.00	0.00	-0.59	-1.00
10.4 (at.%)	0.00	-0.55	-1.07	-0.85
12.5 (at.%)	0.00	-1.11	-0.77	-0.57

Table S4. Energy differences between the CO adsorption on the structures produced by the off-grid and on-grid methods. The positive energies mean that after CO adsorption, the structures determined from on-grid method become more stable, reversing the stabilities from the clean surfaces.

$C_{\rm OV}$ \ $C_{\rm cr}$	0.00 (ML)	0.33 (ML)	0.67 (ML)	1.00 (ML)
0.0 (at.%)	0.00	0.00	0.00	0.00
2.1 (at.%)	0.00	0.00	0.00	0.00
4.2 (at.%)	0.00	0.00	0.79	1.31
6.3 (at.%)	0.00	0.00	0.55	1.04
8.3 (at.%)	0.00	0.00	0.86	1.61
10.4 (at.%)	0.00	0.92	1.40	1.17
12.5 (at.%)	0.00	1.03	0.80	0.76

We find that CO-induced stabilization effect for on-grid structures outweighs the energetic favourability of the off-grid-derived surface, leading to a reversal in the relative stability of the two systems. Firstly, we calculated the energy difference between most stable structures produced by the off-grid and on-grid methods (**Table S3**), showing that the structures identified by the off-grid methods exhibit superior stabilities compared to those identified by the on-grid methods. Then we investigated the energy difference between the total energy of CO adsorption on these two types of structures, as summarized in **Table S4**. Our findings demonstrate that upon CO adsorption, the on-grid structures exhibit greater stabilities than the off-grid structures.

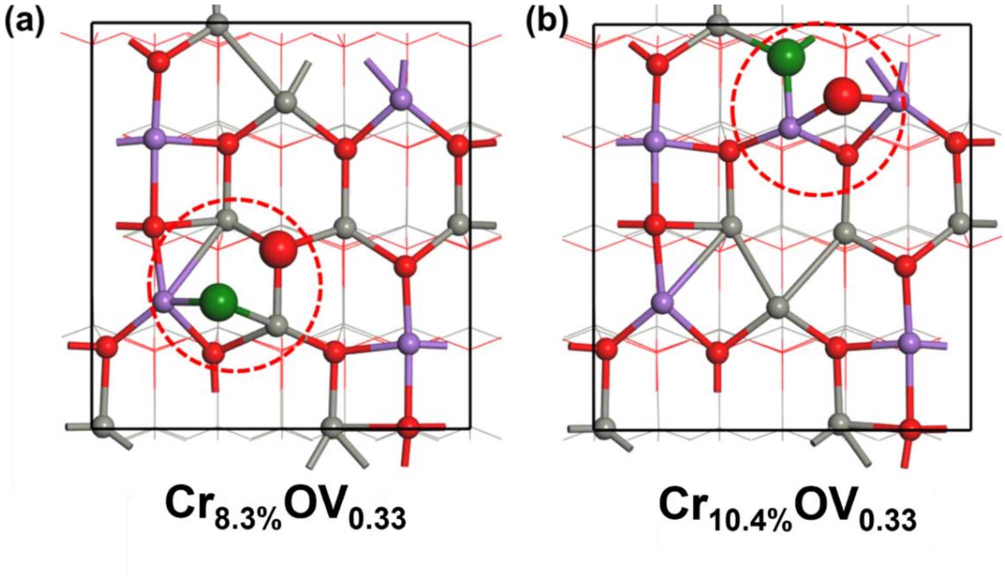

Figure S12. Optimized transition states of C-O bond dissociation on ZnO[$10\bar{1}0$] surfaces with $Cr_{8.3\%}OV_{0.33}$ (a) and $Cr_{10.4\%}OV_{0.33}$ (b).

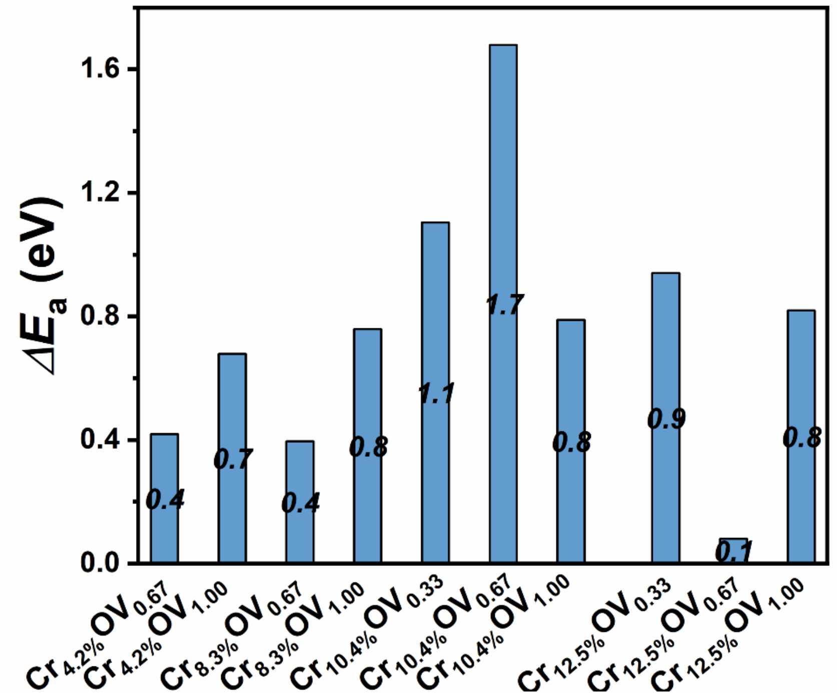

Figure S13. Effective energy barrier(E_a) differences of the C-O bond dissociation on the structures identified by the off-grid and on-grid structure search methods.

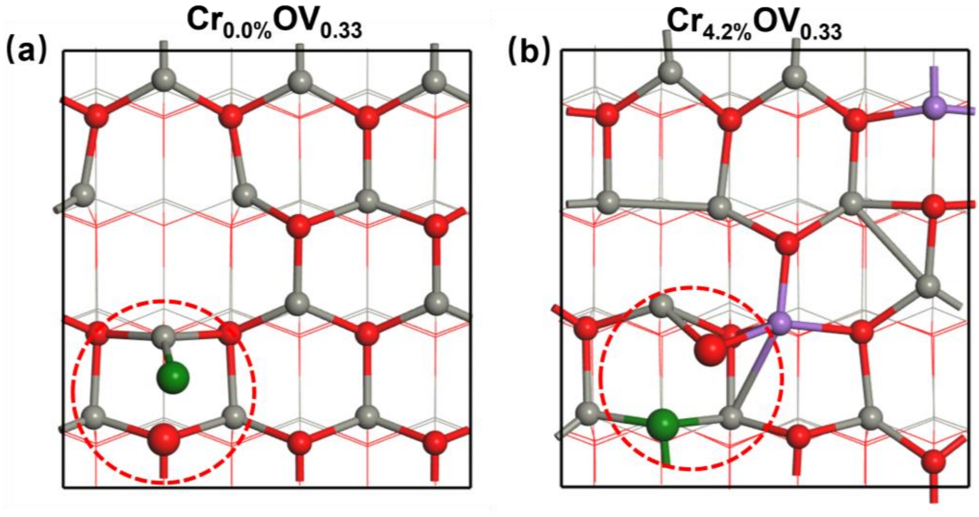

Figure S14. Transition states of C-O bond dissociation on the ZnO surfaces with $Cr_{0.0\%}OV_{0.33}$ (a) and $Cr_{4.2\%}OV_{0.33}$ (b).

It can be seen from **Figure 8e** in the main text that E_a does not fit into a perfect linear relationship with E_{OV}. Especially, for some grey points that covers a range of $\mathcal{C}_{Cr}$ from 0 to 8.3% with 0.33 ML OVs, as $\mathcal{C}_{Cr}$ increases, E_{OV} decreases (see **Figure 4b** in the main text). This is because the surfaces with higher $\mathcal{C}_{Cr}$ possess the ability to reduce the barrier for C-O bond dissociation: the surfaces with higher $\mathcal{C}_{Cr}$ provide more opportunities for the cleavage products to be stabilized by the presence of Cr ions on the surfaces (**Figure S14**).

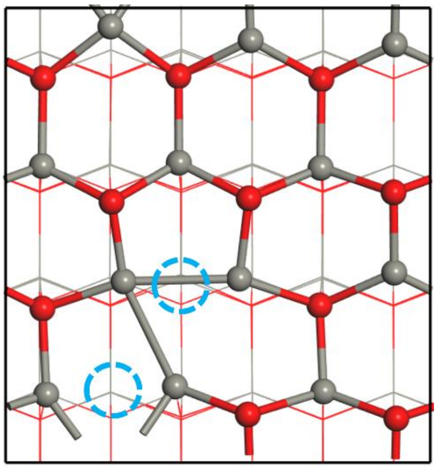

Figure S15. Configuration with a connected 3-coordinated surface OV and a 4-coordinated subsurface OV. The blue dashed circle indicates the OV.

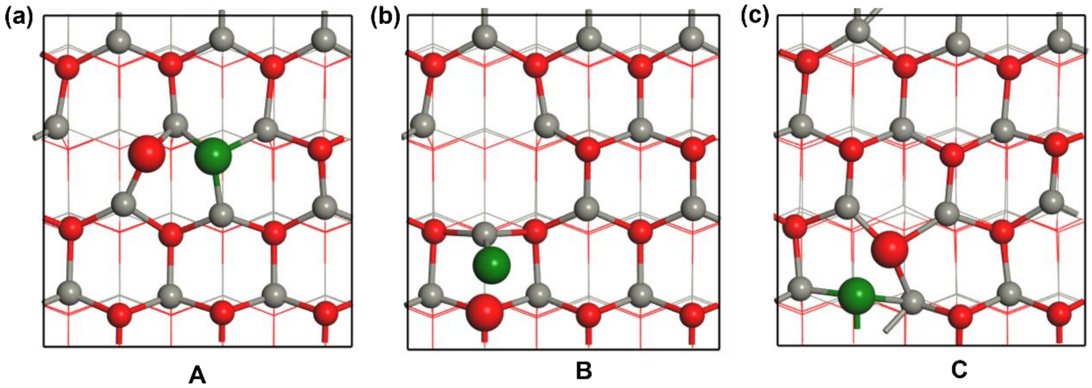

Figure S16. Optimized transition state of C-O bond dissociation on ZnO[$10\bar{1}0$] surfaces with various arrangements of OVs**.**

4. References

1 Johnson, R., Computational Chemistry Comparison and Benchmark Database, NIST Standard Reference Database 101; *NIST*: **2002**.

2 Jiao, F.; Li, J.; Pan, X.; Xiao, J.; Li, H.; Ma, H.; Wei, M.; Pan, Y.; Zhou, Z.; Li, M. J. S., Selective Conversion of Syngas to Light Olefins. *Science.* **2016,** *351* (6277), 1065-1068.

3 Stukowski, A., Visualization and Analysis of Atomistic Simulation Data with Ovito−the Open Visualization Tool. *Model. Simul. Mater. Sci. Eng.* **2010**, *18*, No.015012.
