# Supplementary material for: Unravelling the Impact of Metal Dopants and Oxygen Vacancies on Syngas Conversion over Oxides: A Machine Learning-Accelerated Study of CO Activation on Cr-Doped ZnO Surfaces
Source: ACS Catal. 2023 Nov 8;13(22):15074–86. doi: 10.1021/acscatal.3c03648 (PMC10660660; doi:10.1021/acscatal.3c03648)
Supplement: Supplementary file 2 — cs3c03648_si_002.zip [file cs3c03648_si_002.zip › CIF files/Statement regarding CIF files.docx]

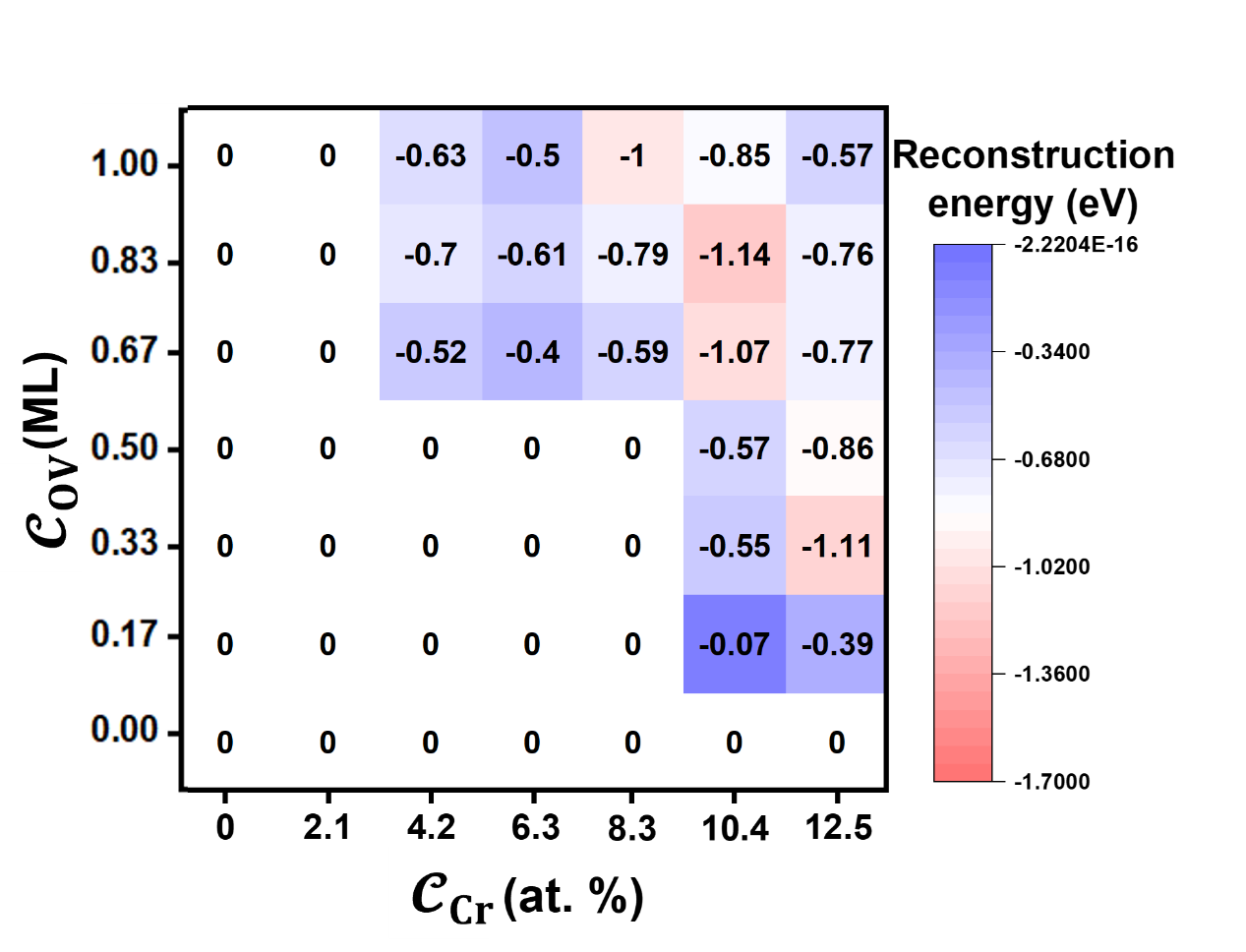


Energy differences between the most stable structures produced by the off-grid and on-grid methods. The number 0 implies that the most stable structures identified by both methods are the same. Consequently, we are providing the CIF files for the unreconstructed structures containing all of the$\mathcal{C}_{\mathrm{Cr}}$ and $\mathcal{C}_{\mathrm{OV}}$, as well as CIF files for the reconstructed structures with a non-zero reconstruction energy.
